# Integrated bioinformatics analysis for the screening of hub genes and therapeutic drugs in ovarian cancer

**DOI:** 10.1186/s13048-020-0613-2

**Published:** 2020-01-27

**Authors:** Dan Yang, Yang He, Bo Wu, Yan Deng, Nan Wang, Menglin Li, Yang Liu

**Affiliations:** 1 Department of Environmental Health, School of Public Health, China Medical University, 77th Puhe Road, Shenyang, 110122 Liaoning China; 2 Department of Central Laboratory, The First Affiliated Hospital, China Medical University, 155th Nanjing North Street, Shenyang, 110001 Liaoning China; 3 Department of Anus and Intestine Surgery, The First Affiliated Hospital, China Medical University, 155th Nanjing North Street, Shenyang, 110001 Liaoning China

**Keywords:** Ovarian cancer, Differentially expressed genes, Functional enrichment analysis, Protein-protein interaction, Survival analysis, miRNA-hub gene network

## Abstract

**Background:**

Ovarian cancer (OC) ranks fifth as a cause of gynecological cancer-associated death globally. Until now, the molecular mechanisms underlying the tumorigenesis and prognosis of OC have not been fully understood. This study aims to identify hub genes and therapeutic drugs involved in OC.

**Methods:**

Four gene expression profiles (GSE54388, GSE69428, GSE36668, and GSE40595) were downloaded from the Gene Expression Omnibus (GEO), and the differentially expressed genes (DEGs) in OC tissues and normal tissues with an adjusted *P-value* < 0.05 and a |log fold change (FC)| > 1.0 were first identified by GEO2R and FunRich software. Next, Gene Ontology (GO) and Kyoto Encyclopaedia of Genes and Genomes (KEGG) analyses were performed for functional enrichment analysis of these DEGs. Then, the hub genes were identified by the cytoHubba plugin and the other bioinformatics approaches including protein-protein interaction (PPI) network analysis, module analysis, survival analysis, and miRNA-hub gene network construction was also performed. Finally, the GEPIA2 and DGIdb databases were utilized to verify the expression levels of hub genes and to select the candidate drugs for OC, respectively.

**Results:**

A total of 171 DEGs were identified, including 114 upregulated and 57 downregulated DEGs. The results of the GO analysis indicated that the upregulated DEGs were mainly involved in cell division, nucleus, and protein binding, whereas the biological functions showing enrichment in the downregulated DEGs were mainly negative regulation of transcription from RNA polymerase II promoter, protein complex and apicolateral plasma membrane, and glycosaminoglycan binding. As for the KEGG-pathway, the upregulated DEGs were mainly associated with metabolic pathways, biosynthesis of antibiotics, biosynthesis of amino acids, cell cycle, and HTLV-I infection. Additionally, 10 hub genes (KIF4A, CDC20, CCNB2, TOP2A, RRM2, TYMS, KIF11, BIRC5, BUB1B, and FOXM1) were identified and survival analysis of these hub genes showed that OC patients with the high-expression of CCNB2, TYMS, KIF11, KIF4A, BIRC5, BUB1B, FOXM1, and CDC20 were statistically more likely to have poorer progression free survival. Meanwhile, the expression levels of the hub genes based on GEPIA2 were in accordance with those based on GEO. Finally, DGIdb database was used to identify 62 small molecules as the potentially targeted drugs for OC treatment.

**Conclusions:**

In summary, the data may produce new insights regarding OC pathogenesis and treatment. Hub genes and candidate drugs may improve individualized diagnosis and therapy for OC in future.

## Introduction

Ovarian cancer (OC) ranks fifth as a cause of gynecological cancer-associated deaths globally [1], with an estimated 238,700 new cases and 151,900 deaths in 2012 [2]. There are many risk factors associated with OC, such as a family history of breast cancer or OC, excess body weight, smoking, earlier menstruation or later menopause, and not giving birth [2]. Due to the vague symptoms, more than 70% of OC cases are diagnosed at an advanced stage [3]. In most countries, the 5-year survival rate of OC is usually lower than 40% [4]. The poor prognosis and high mortality can be mainly attributed to the lack of early and effective detection methods [5]. Therefore, more efforts need to be invested towards the identification and understanding of novel biomarkers and specific targets of OC, which is considered the key to developing more effective diagnostic and therapeutic strategies.

Recently, gene profiles and gene chips have been used to screen differentially expressed genes (DEGs) [6]. Reanalyzing these data can provide new insights into current research on OC [7]. However, current studies on biomarkers may be insufficient, and the DEG results may be inconsistent because of the complex tumor heterogeneity and complicated molecular regulatory mechanism of OC. Furthermore, chemotherapy resistance remains a major challenge and causes most failures of treatment and the short overall survival (OS) and progression free survival (PFS) of OC patients. Many bioinformatical studies on OC have been proven to be effective and reliable [7, 8], which means integrated bioinformatics analysis could assist with exploring the biomarkers and the mechanisms underlying the tumorigenesis and progression of cancer.

In this study, four original gene expression profiles (GSE54388, GSE69428, GSE36668, and GSE40595) were downloaded from the Gene Expression Omnibus (GEO) database. DEGs were first screened based on the above four datasets. Subsequently, integrated bioinformatics analyses including Gene Ontology (GO) term analysis, Kyoto Encyclopedia of Genes and Genomes (KEGG) pathway enrichment analysis, protein-protein interaction (PPI) construction, and the identification of hub genes were performed. Furthermore, survival analysis and validation of the hub genes were also performed. Finally, the miRNA-hub gene network was constructed, and targeted small molecular drugs for OC were identified.

## Materials and methods

### Data sources

The NCBI-GEO database is a free and public database containing gene profiles. Four microarray datasets (GSE54388, GSE69428, GSE36668, and GSE40595) were obtained from the GEO database (https://www.ncbi.nlm.nih.gov/gds/). The inclusion criteria for the above gene expression profiles were set as follows: (1) the tissue samples were obtained from human OC tissues and normal tissues; (2) the number of samples in each dataset was more than 8; (3) and all these profiles were based on GPL570 (Affymetrix Human Genome U133 Plus 2.0 Array).

### Identification of DEGs

GEO2R is an interactive web tool that can compare and analyze two different groups of samples under the same experimental conditions [9]. In this study, the selected datasets of OC tissues and normal tissues were first analyzed by GEO2R. Subsequently, the analyzed results were downloaded in Microsoft Excel format, and genes that met the cutoff criteria of the adjusted *P-value* < 0.05 and |log fold change (FC)| > 1.0 were considered DEGs [8]. Finally, the FunRich tool (version 3.1.3) was applied to illustrate the intersection of the DEGs. Additionally, an online tool, ClustVis, was used to draw the heatmap of the DEGs [10]. Specifically, the gene expression matrix for the heatmap was derived from GSE69428.

### Functional enrichment analysis of DEGs

GO functional analysis and KEGG pathway analysis were carried out to predict the potential functions of the DEGs by using the Database for Annotation, Visualization and Integrated Discovery (DAVID; https://david.ncifcrf.gov/; version 6.8). Upregulated and downregulated DEGs were submitted to the DAVID online program. The top 10 items of the cellular component (CC), biological process (BP), and molecular function (MF) categories and KEGG pathways were then sorted and presented in the form of bubble maps. These bubble plots were drawn using the ggplot2 R package based on *P-value* through the statistical software R (version 3.6.1). *P < 0.05* was considered statistically significant.

### PPI network construction and module analysis

The PPI network of the identified DEGs was constructed by an online tool, the Search Tool for the Retrieval of Interacting Genes/Proteins (STRING; https://string-db.org/), with an interaction score > 0.4. The active interaction sources included text mining, experiments, databases, coexpression, neighborhood, gene fusion, and corecurrence. Furthermore, the interaction network between the DEGs and their related genes was presented with the minimum number of interactions = 2. Subsequently, the modules of the PPI network were screened by the Cytoscape software (version 3.7.1) plugin Molecular Complex Detection (MCODE) with default parameters as follows: degree cutoff = 2, node score cutoff = 0.2, k-score = 2, and max. Depth = 100. In this study, the criteria of the top four modules were set with MCODE scores ≥2.8 and nodes ≥3. KEGG pathway analysis of the genes in each module was performed by DAVID. Finally, based on highest degree of connectivity, the top 10 genes were selected as the target hub genes by using the Cytoscape plugin cytoHubba. The construction of the PPI network and coexpression analysis of the hub genes were performed by STRING. The criteria of the PPI network included a confidence score ≥ 0.4 and a maximum number of interactions ≤5.

### Survival analysis and validation of the hub genes

The Kaplan-Meier plotter can assess the prognostic effect of genes on survival in many types of cancer, including 6234 breast, 2190 ovarian, 3452 lung, and 1440 gastric cancer samples (http://kmplot.com/analysis/). Patients with OC were categorized into two groups, namely, a high-expression group and a low-expression group, according to the expression of a particular gene. The general inclusion criteria for the survival analysis were set as follows: (1) used only the JetSet best probe set; (2) used a 2017 version dataset; (3) excluded biased arrays. Then, the PFS was analyzed for the above two groups for each hub gene for the OC patients and the serous OC (SOC) patients for “all stages”, “early stages (stages 1 and 2)”, and “advanced stages (stages 3 and 4)”. These analyses are shown in the form of a survival prognosis forest map and survival curves according to the hazard ratio (HR), 95% confidence interval (95% CI), and log-rank *P-value.* The forest map was constructed by STATA version 15.0 (StataCorp, College Station, Texas, USA).

Additionally, the expression levels of the hub genes between OC and normal samples were verified by Gene Expression Profiling Interactive Analysis (GEPIA2; http://gepia2.cancer-pku.cn/#index). Then, GEPIA2 was also used to explore the variations among OC samples at different stages. ANOVA was used to assess the statistical significance of the variations. Pr (>F) < 0.05 was considered statistically significant. Furthermore, the cBio Cancer Genomics Portal (https://www.cbioportal.org/; version 3.0.2) online tool was used to present the genetic alteration information of the hub genes.

### miRNA-hub gene network construction

The Encyclopedia of RNA Interactomes (ENCORI) is an open-source platform mainly focusing on miRNA-target interactions (http://starbase.sysu.edu.cn/; version 3.0). ENCORI utilizes eight established miRNA-target prediction databases, including PITA, RNA22, miRmap, microT, miRanda, PicTar, TargetScan, and pancancerNum. In this study, miRNAs were considered the targeted miRNAs of hub genes based on at least two databases being selected among the following databases: miRanda, PITA, PicTar, and TargetScan [11]. Subsequently, the coexpression network of the hub genes and their targeted miRNAs was visualized by Cytoscape software.

### Drug-hub gene interaction

Drugs were selected based on the hub genes that served as promising targets by using the Drug-Gene Interaction Database (DGIdb; http://www.dgidb.org/search_interactions; version 3.0.2 – sha1 ec916b2). In this study, the final drug list included only drugs that were approved by the Food and Drug Administration (FDA). The online tool named STITCH (http://stitch.embl.de/cgi/input.pl?UserId=E40G4aCOYHFw&sessionId=8fGboRzTdag6; version 5.0) was applied to construct the interaction network between the potential drugs and the hub genes [11].

## Results

### Identification of DEGs in OC

**Fig. 1:**
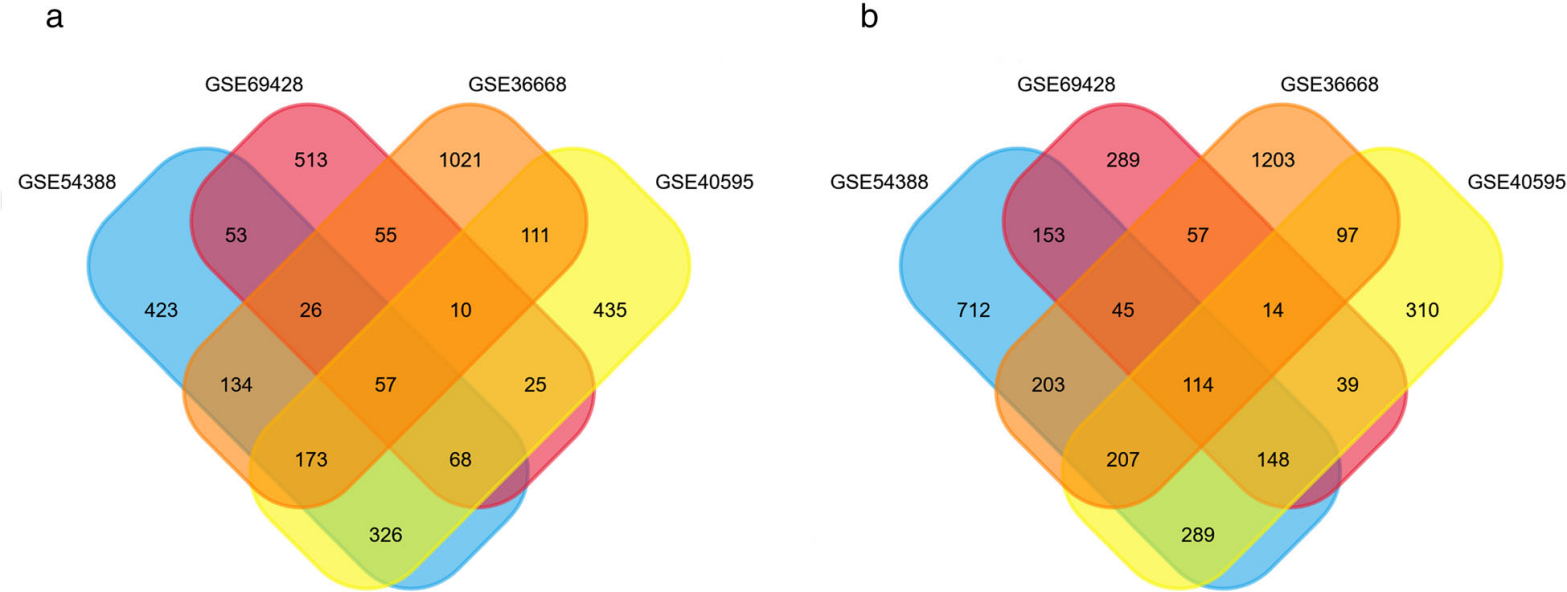
Venn diagram for overlapping differentially expressed genes (DEGs) based on datasets. The intersection of downregulated (**a**) and upregulated (**b**) DEGs was identified from the four datasets, namely, GSE54388, GSE69428, GSE36668, and GSE40595

Four expression profiles (GSE54388, GSE69428, GSE36668, and GSE40595) were obtained from the GEO database. The specific details of the above datasets are presented in Table 1. In this study, the microarray datasets were all based on the GPL570 platform (Affymetrix Human Genome U133 Plus 2.0 Array). Three of the datasets were from SOC, and GSE40595 was from OC stroma (Table 1). GSE54388 consisted of 16 cases and 6 controls; GSE69428 contained 10 cases and 10 controls; GSE36668 included 4 cases and 4 controls; and GSE40595 contained 63 cases and 14 controls (Table 1). The Venn diagrams indicated that a total of 171 DEGs were identified from the four microarray profile datasets, including 114 upregulated genes and 57 downregulated genes in OC tissues compared to normal controls (Fig. 1 and Table 2). In addition, the expression levels of these DEGs are visualized in the form of a heatmap in Additional file 1: Figure S1.

**Table 1:** Detailed information on the GEO microarray profiles of OC patients

No. of GEO profile	Type	Source	Case	Control	Platform	Annotation platform
GSE54388	mRNA	serous ovarian cancer	16	6	GPL570	Affymetrix Human Genome U133 Plus 2.0 Array
GSE69428	mRNA	serous ovarian cancer	10	10	GPL570	Affymetrix Human Genome U133 Plus 2.0 Array
GSE36668	mRNA	serous ovarian cancer	4	4	GPL570	Affymetrix Human Genome U133 Plus 2.0 Array
GSE40595	mRNA	ovarian cancer stroma	63	14	GPL570	Affymetrix Human Genome U133 Plus 2.0 Array

*GEO* Gene Expression Omnibus

**Table 2:** Information on the overlapping DEGs screened from the datasets

DEGs	Gene name
Upregulated (114)	WHSC1, VEGFA, TYMS, TSTA3, TPI1, TPD52L2, TOP2A, TNNT1, TMPRSS4, TIMELESS, C20orf24, TBC1D7, TACC3, STC2, SORT1, BOLA2, SLC52A2, SLC4A11, SLC39A4, SLC38A1, SLC2A1, SLC19A1, SHMT2, SCRIB, SAC3D1, S100A2, RRM2, RNASEH2A, RACGAP1, PXDN, PUF60, PTTG1, PSAT1, PPP1R14B, PIK3R3, PBK, OVOL2, NR2F6, NOV, NDUFB9, NDC80, NCAPG, MYCL, MUC16, MT1F, MRPS15, MRPL12, MLF2, MCM4, MCAM, MAL, LSM4, LPCAT1, C1orf186, LAPTM4B, KLK8, KLK7, KLK6, KLHL14, KIF4A, KIF11, KIAA0101, ISG15, IRAK1, IDH2, HN1, HMGB3, HMGA1, H2AFX, GPSM2, FOXM1, FGF18, FEN1, FDPS, ESRP1, EPT1, ENO1, E2F8, DYRK2, DTL, DSC2, DPP3, DHCR24, DGAT1, DDX39A, DBN1, CYC1, CTPS1, CRABP2, COPE, COL4A1, CLPTM1L, CLDN3, CHD7, CDH6, CDCA3, CDC20, CD47, CCNE1, CCNB2, C9orf16, C1orf106, BUB1B, BOLA2B, BIRC5, BAK1, AURKAIP1, ATP6V1B1, ASS1, ARL4C, ARF3, APOA1, AIF1L, ABHD11
Downregulated (57)	ZEB2, ZBTB20, WHAMMP2, WHAMMP3, WDR17, TLE4, THBD, TFPI, SYTL3, SNCAIP, SMIM14, SLC4A4, SCD5, RUNX1T1, RUNDC3B, RNASE4, PTPN13, PRKAR1A, PLCL2, PIP5K1B, PDK4, PDGFRA, NDNF, NBEA, N4BP2L2, N4BP2L1, MPDZ, MITF, MEF2C, MAF, FAM153A, FAM153B, FAM153C, KLHDC1, KLF2, KAT2B, KANSL1L, ITM2A, IL6ST, GPRASP1, GNG11, FOXP2, DPYD, DPY19L2P2, DMD, DCN, CXorf57, CIRBP, CHGB, CELF2, CDC14A, BNC2, ARMCX1, ALDH1A1, ABCA8, ABCA5, AASS

A total of 171 DEGs were identified from the four gene expression profiles, including 114 upregulated DEGs and 57 downregulated DEGs in OC tissues compared to normal tissues

### Functional enrichment analysis of DEGs

**Fig. 2:**
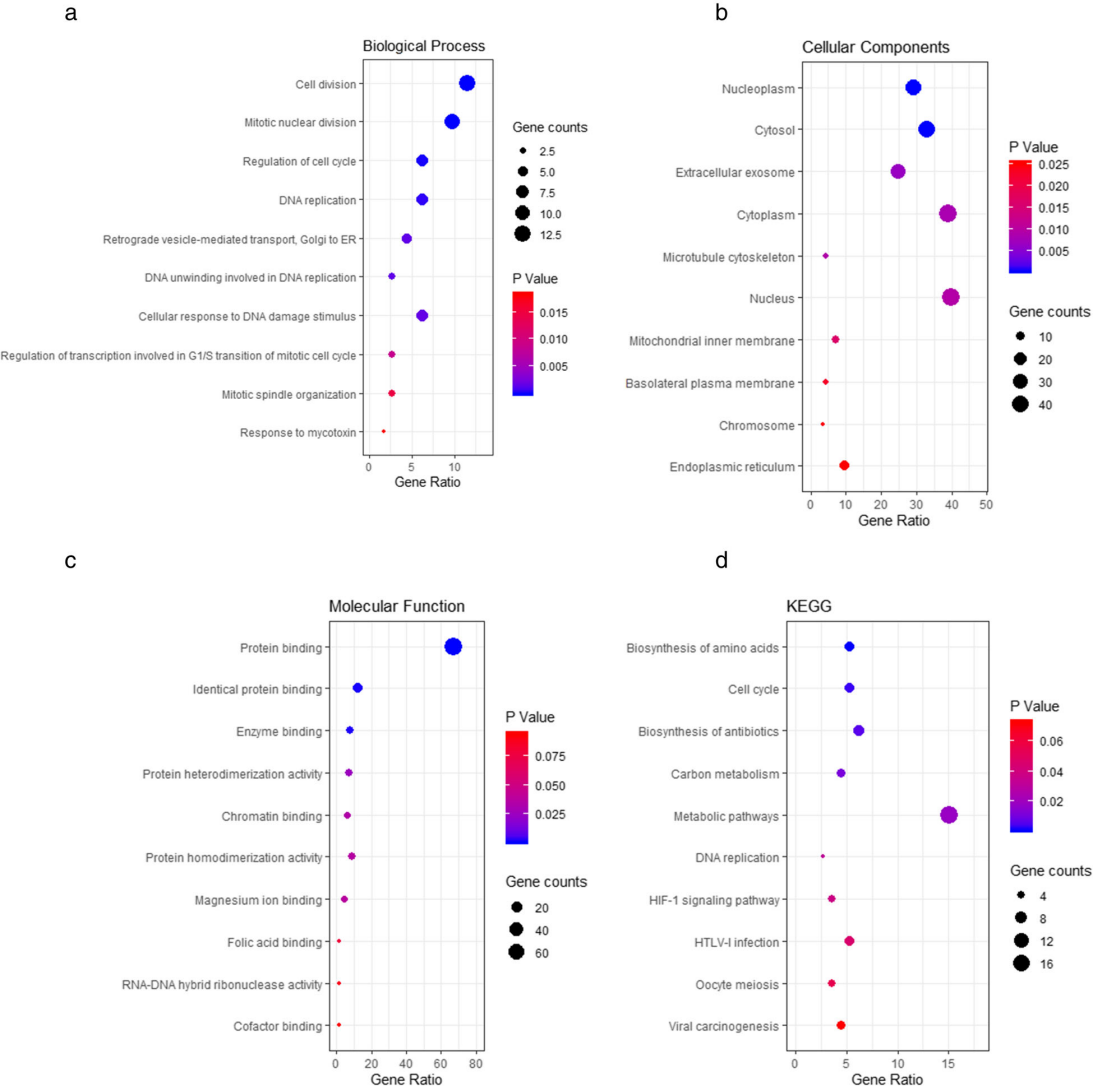
Bubble map for GO and KEGG pathway analyses of upregulated DEGs. The top 10 items of the GO and KEGG pathway enrichment analyses are illustrated in the form of a bubble plot using the ggplot2 package for R software. A *P-value* < 0.05 was considered statistically significant. **a** biological processe, **b** cellular components, **c** molecular function, and **d** KEGG pathways. GO, Gene Ontology; KEGG, Kyoto Encyclopedia of Genes and Genomes

A total of 114 upregulated genes and 57 downregulated genes were analyzed by DAVID software. The top 5 significant terms from the GO enrichment analysis showed that in the BP category, the upregulated DEGs were involved in cell division, mitotic nuclear division, regulation of the cell cycle, DNA replication, and cellular response to DNA damage stimulus (Fig. 2a), whereas the downregulated DEGs were significantly involved in the negative regulation of transcription from the RNA polymerase II promoter, the positive regulation of transcription from the RNA polymerase II promoter, skeletal muscle tissue development, the viral process, and the regulation of phosphatidylinositol 3-kinase signaling (Additional file 2: Table S1). For the CC category, the upregulated DEGs were correlated with the nucleus, cytoplasm, cytosol, nucleoplasm, and extracellular exosomes (Fig. 2b), whereas the downregulated DEGs were associated with the protein complex and apicolateral plasma membrane (Additional file 2: Table S1). For the MF category, the upregulated DEGs were enriched in protein binding, identical protein binding, protein homodimerization activity, enzyme binding, and protein heterodimerization activity (Fig. 2c), whereas the downregulated DEGs were related to glycosaminoglycan binding (Additional file 2: Table S1). For KEGG pathway enrichment analysis, the downregulated DEGs were not enriched, whereas the top five significant KEGG pathways of the upregulated DEGs included metabolic pathways, biosynthesis of antibiotics, biosynthesis of amino acids, the cell cycle, and human T-lymphotropic virus type I (HTLV-I) infection (Fig. 2d). Furthermore, specific information on the upregulated DEGs identified in each category in the functional enrichment analysis are presented in Additional file 3: Table S2.

### PPI network construction and module analysis

The PPI network of the DEGs in OC was constructed based on the information obtained from the STRING database. When 171 DEGs were submitted to the STRING database, there were 3 unidentified gene IDs, namely, WHAMMP2, WHAMMP3, and FAM153C. Subsequently, 168 genes were mapped into the PPI network. The PPI network included 168 nodes and 450 edges, and its PPI enrichment *P-value* was lower than 1.0e − 16 (Additional file 4: Figure S2). In addition, an interaction network of 168 DEGs and their neighboring genes was constructed through FunRich (Additional file 5: Figure S3). Then, the significant modules were identified via the MCODE plugin. As illustrated in Additional file 6: Figure S4, the top four functional clusters of modules were selected (module 1, MCODE score = 21.826; module 2, MCODE score = 3.333; module 3, MCODE score = 3; and module 4, MCODE score = 2.8). KEGG pathway analysis of each module was performed by DAVID, as shown in Additional file 7: Table S3. As shown in Additional file 6: Figure S4, module 1 consisted of 24 nodes and 251 edges, the average node degree was 20.9, and the PPI enrichment *P-value* was lower than 1.0e − 16 (Additional file 6: Figure S4A). The KEGG pathway analysis of module 1 indicated that these genes were involved in the cell cycle, DNA replication, and oocyte meiosis (Additional file 7: Table S3). Module 2 consisted of 4 nodes and 5 edges (Additional file 6: Figure S4B), with genes enriched in ribosomes (Additional file 7: Table S3). Module 3 contained 3 nodes and 3 edges (Additional file 6: Figure S4C), with genes related to the biosynthesis of amino acids, the HIF-1 signaling pathway, and carbon metabolism (Additional file 7: Table S3). Finally, module 4 comprised 6 nodes and 7 edges (Additional file 6: Figure S4D), with genes associated with fat digestion and absorption and the PPAR signaling pathway (Additional file 7: Table S3). The PPI enrichment *P-value* of each module was lower than 0.05.

**Fig. 3:**
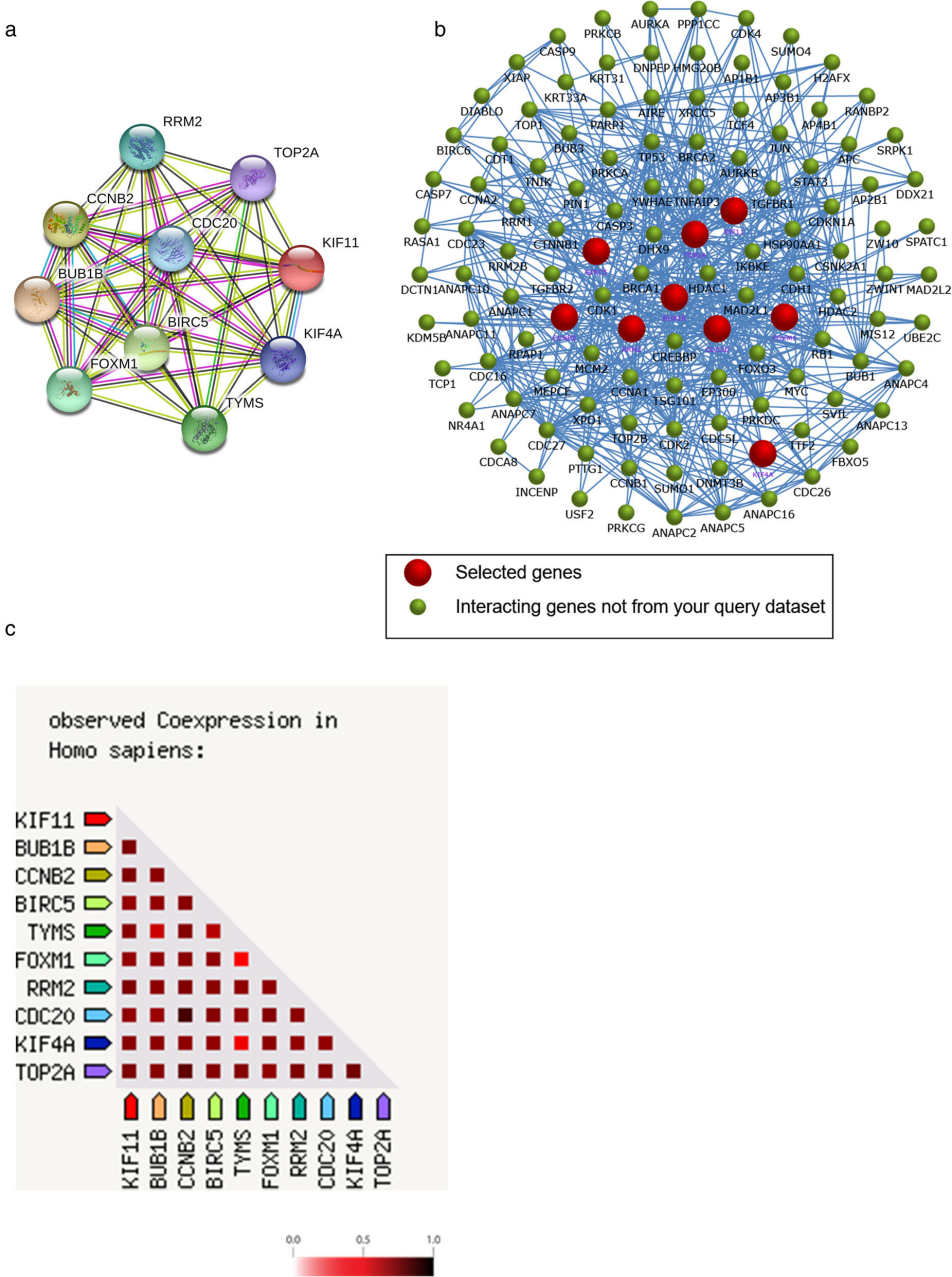
PPI network and coexpression analysis of the hub genes in OC. **a** The PPI network of the hub genes using the STRING online database. **b** The interaction network of the hub genes and their related neighboring genes using the FunRich software. **c** The coexpression analysis of the hub genes using the STRING online database

Ten genes (TYMS, CCNB2, KIF11, RRM2, CDC20, TOP2A, BUB1B, BIRC5, KIF4A, and FOXM1) with the highest degree scores were identified as the hub genes for OC by applying the cytoHubba plugin (Additional file 8: Table S4). All the hub genes were upregulated DEGs, as shown in Table 2. Additionally, the STRING online database was used to construct the PPI network of the hub genes (Fig. 3a), and FunRich software was used to draw the interaction network of the hub genes and their related genes (Fig. 3b). As shown in Fig. 3a, the PPI network of the hub genes consisted of 10 nodes and 45 edges, while its average local clustering coefficient was 1, and its PPI enrichment P-value was lower than 1.0e − 16. Additionally, the results of the gene coexpression analysis of the ten hub genes showed that these hub genes might actively interact with each other (Fig. 3c).

### Survival analysis, genetic information and hub gene expression

**Fig. 4:**
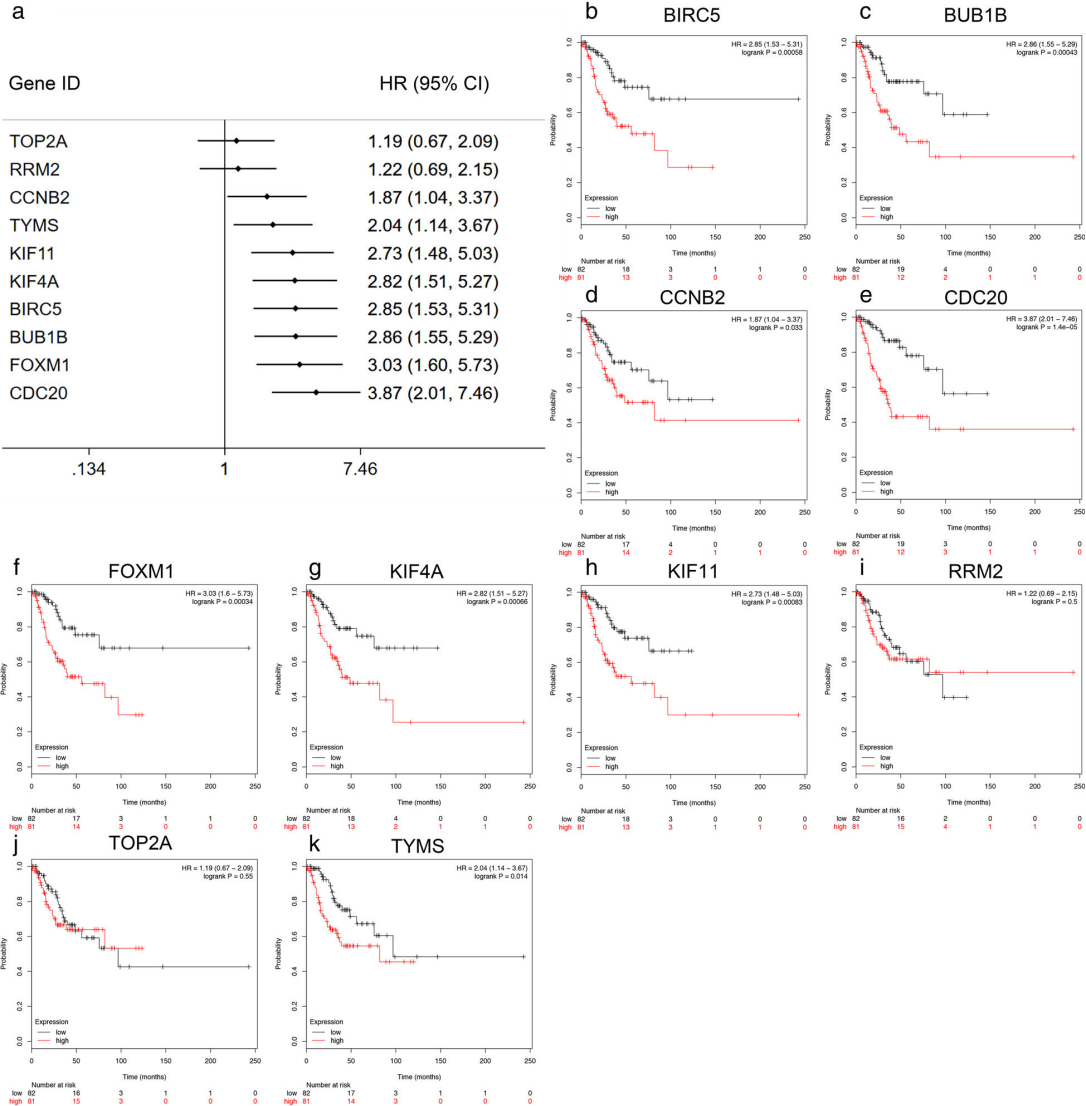
Progression free survival analyses of hub genes in patients with stage 1 or 2 OC. **a** Survival prognosis forest map of the hub genes related to prognosis in OC patients. Each point in the forest plot represents the hazard ratio (HR) of the gene, and the line on both sides of the point represents the 95% confidence interval (95% CI). **b**-**k** Survival curves were constructed by the Kaplan-Meier plotter online database based on the low and high expression of the hub genes in OC patients. Log-rank *P* < 0.05 was considered statistically significant

The prognostic information of these 10 hub genes for OC and SOC at different stages was analyzed by the Kaplan-Meier plotter database. A survival prognosis forest map of these genes in early stage (Stages 1 and 2) is shown in Fig. 4a, and the survival curves of these genes are presented in Fig. 4b-k. Among the 10 hub genes, 8 hub genes were significantly associated with the PFS of OC patients at an early stage (stages 1 and 2) (Fig. 4). Except for TOP2A [HR = 1.19 (0.67–2.09), *P* = 0.55] (Fig. 4j) and RRM2 [HR = 1.22 (0.69–2.15), *P* = 0.50] (Fig. 4i), early-stage OC patients with higher expression levels of CCNB2 [HR = 1.87 (1.04–3.37), *P* = 0.033] (Fig. 4d), TYMS [HR = 2.04 (1.14–3.67), *P* = 0.014] (Fig. 4k), KIF11 [HR = 2.73 (1.48–5.03), *P* < 0.001] (Fig. 4h), KIF4A [HR = 2.82 (1.51–5.27), *P* < 0.001] (Fig. 4g), BIRC5 [HR = 2.85 (1.53–5.31), *P* < 0.001] (Fig. 4b), BUB1B [HR = 2.86 (1.55–5.29), *P* < 0.001] (Fig. 4c), FOXM1 [HR = 3.03 (1.60–5.73), *P* < 0.001] (Fig. 4f), and CDC20 [HR = 3.87 (2.01–7.46), *P* < 0.001] (Fig. 4e) were significantly related to poorer PFS (Fig. 4). For SOC patients at stages 1 and 2, except for RRM2, FOXM1, and TOP2A, the remaining 7 hub genes presented statistically significant trends (Additional file 9: Figure S5). In addition, the relationship between PFS and the expression of these 10 hub genes in OC and SOC patients in “all stages” and “advanced stages” are presented in Additional file 10: Figures S6, Additional file 11: Figure S7, Additional file 12: Figure S8, and Additional file 13: Figure S9. More interestingly, increased expression of KIF4A and TYMS were related to poorer PFS in early stage (stages 1 and 2) OC and SOC patients (Fig. 4g, k, Additional file 9: Figure S5G & K), whereas decreased expression of the above 2 hub genes were associated with better PFS in advanced stage (stages 3 and 4) OC and SOC patients (Additional file 12: Figure S8 and Additional file 13: Figure S9).

**Fig. 5:**
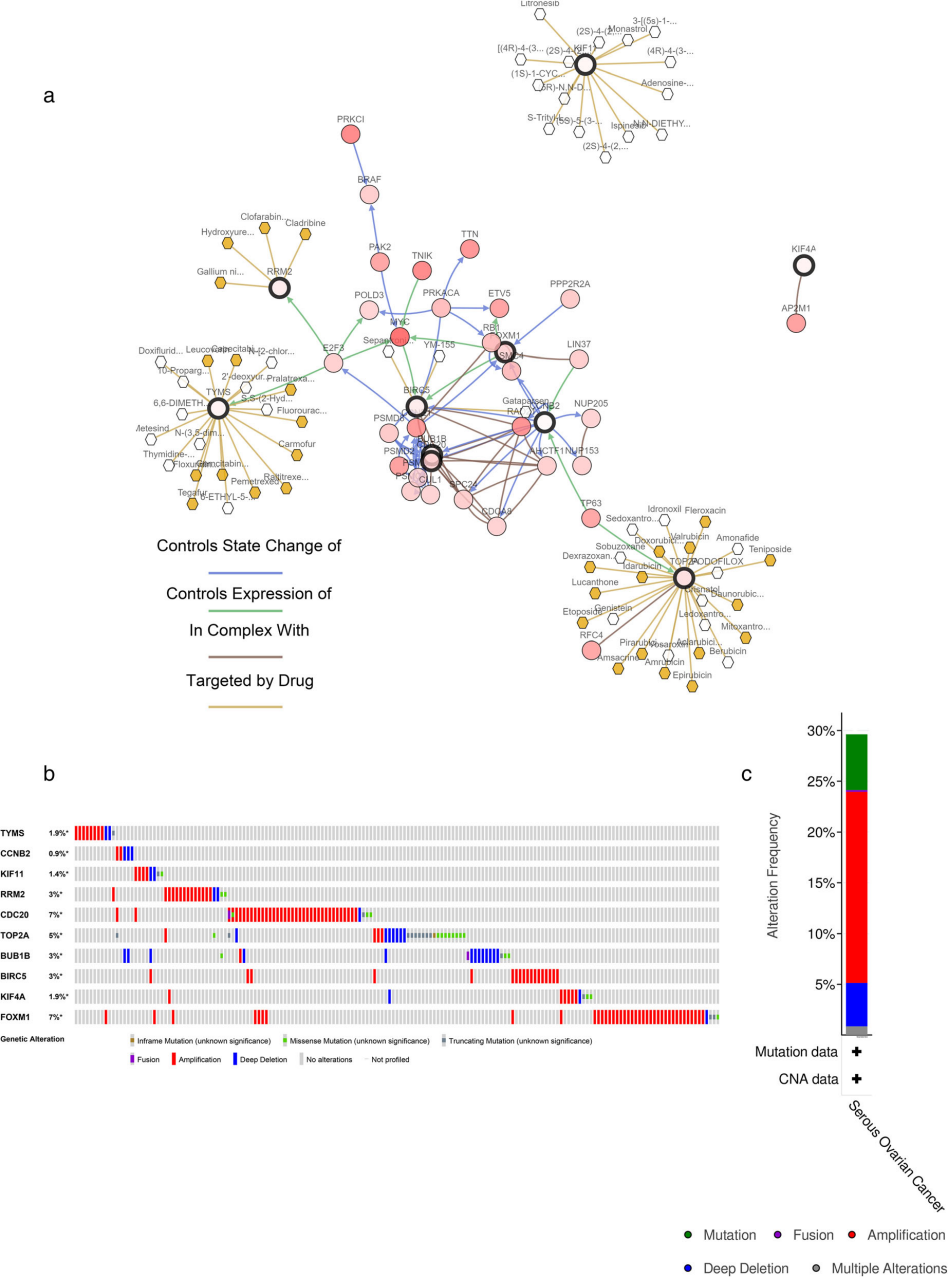
Information on the genetic alterations of the hub genes. **a** The association between the hub genes and related drugs was identified by cBioPortal, and the network of 10 hub genes and the 50 most frequently altered neighboring genes was also constructed. **b** The genetic alterations related to the hub genes are shown through a visual summary across a set of ovarian serous cystadenocarcinoma samples (data from TCGA, PanCancer Atlas). **c** An overview of the alterations of the 10 hub genes in the genomics datasets of ovarian serous cystadenocarcinoma in the TCGA database

Subsequently, cBioPortal was used to determine the genetic alteration information of the 10 hub genes, as illustrated in Fig. 5. The network shown in Fig. 5a consists of 60 nodes, including 10 hub genes and the 50 most frequently altered neighboring genes (out of a total of 767). Drugs targeting the 10 hub genes are also presented in Fig. 5a. From this network, only KIF11, TOP2A, RRM2, TYMS, and BIRC5 are currently considered chemotherapy targets (Fig. 5a). Alteration information of the 10 hub genes is shown in Fig. 5b and c. As presented in Fig. 5b, the hub genes were altered in 173 (30%) queried patients or samples. CDC20 and FOXM1 were altered most often (7 and 7%, respectively). These alterations included amplification, deep deletion, truncating mutation, missense mutation, inframe mutation, and fusion (Fig. 5b). Among the different types of alterations, amplification accounted for the highest percentage (Fig. 5c).

**Fig. 6:**
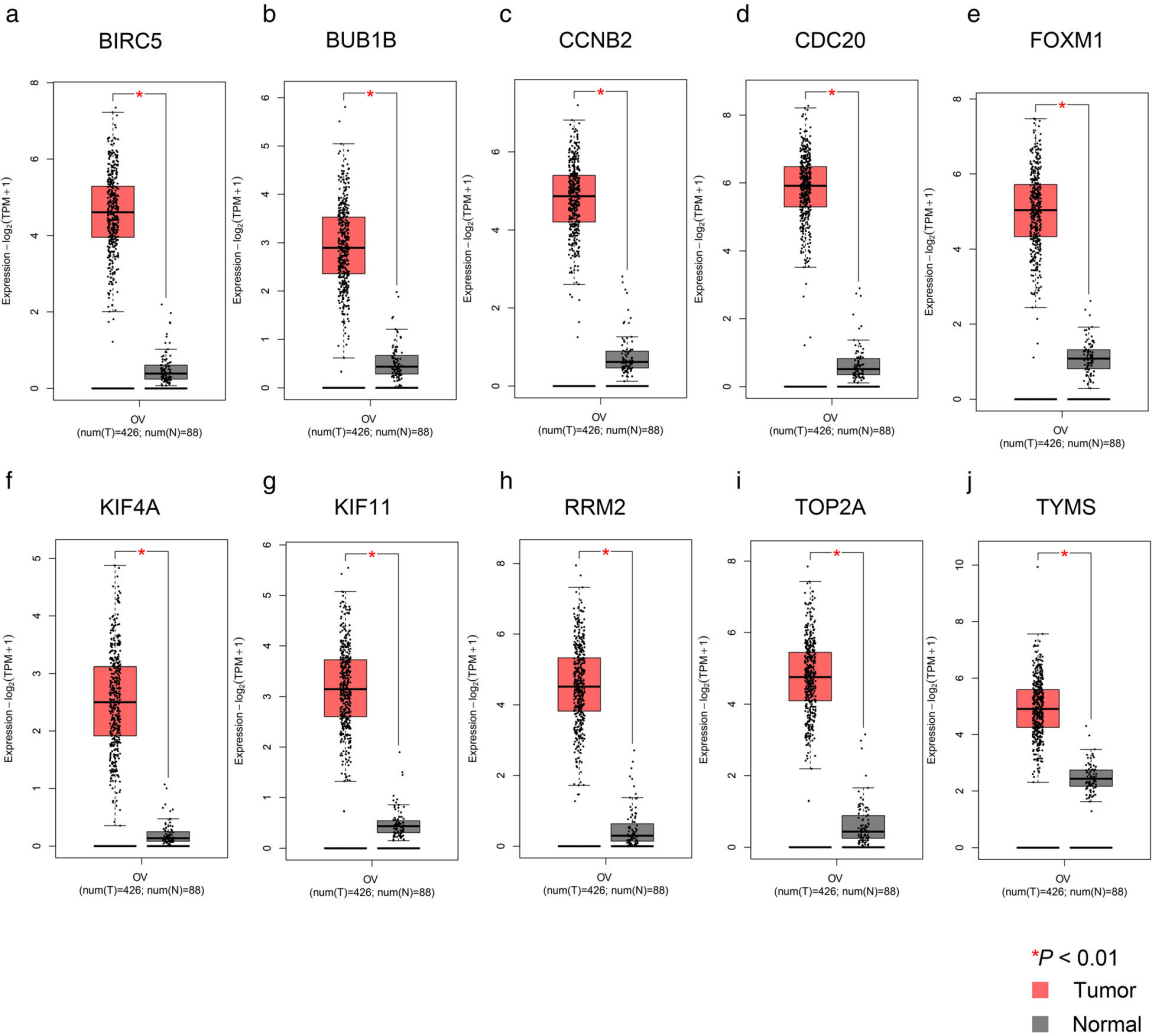
The expression level of hub genes in OC tissues and normal tissues from patients (**a**-**j**). To further verify the expression level of the hub genes between OC tissues and normal tissues, the hub genes were analyzed by the GEPIA2 online database. *P* < 0.01 was considered statistically significant

**Fig. 7:**
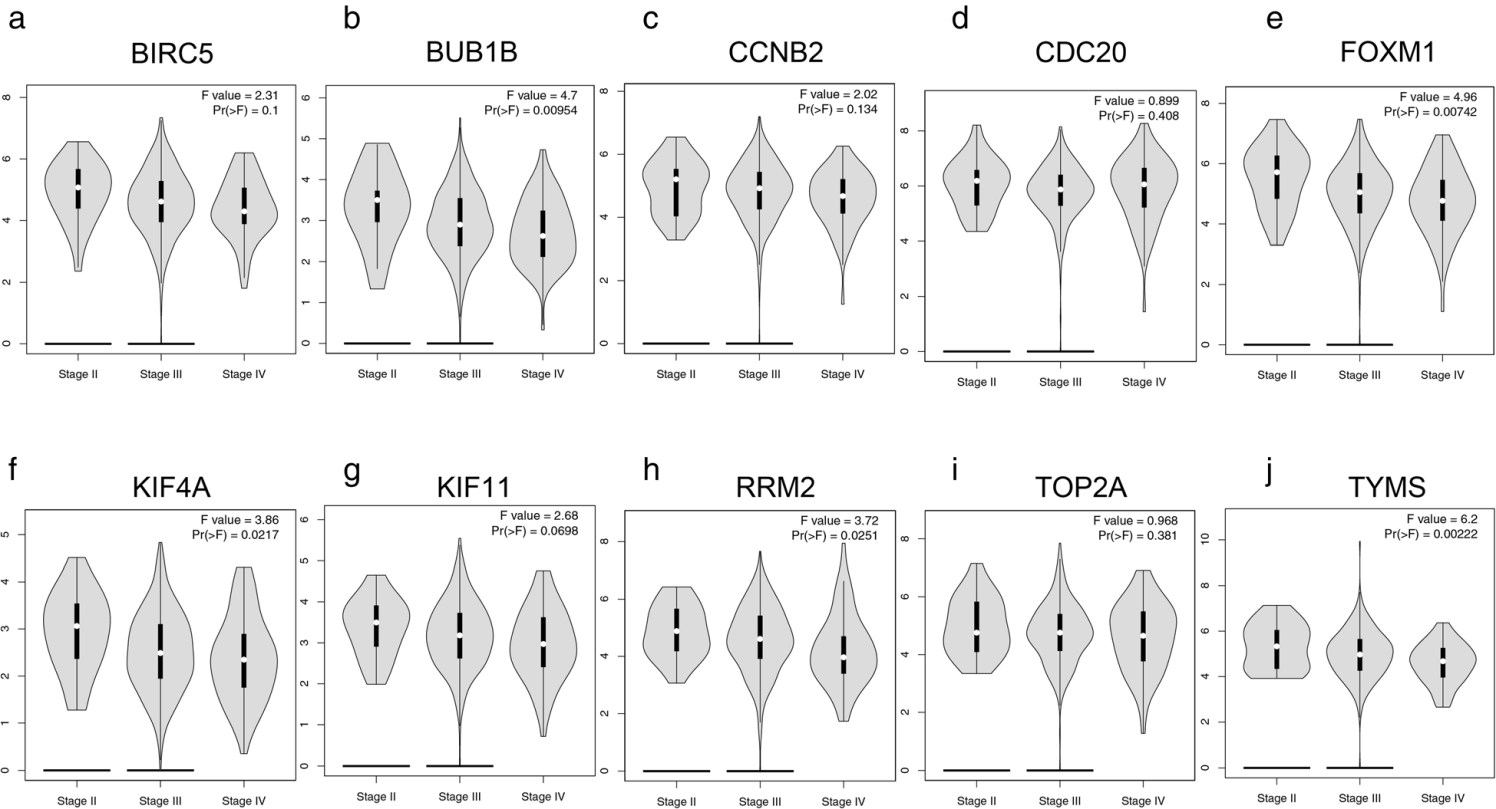
The expression level of hub genes in OC tissues at different stages (**a**-**j**). To further verify the expression level of the hub genes in OC tissues at different stages, the hub genes were analyzed by the GEPIA2 online database. ANOVA was performed to assess the statistical significance of the variations. Pr (>F) < 0.05 was considered statistically significant

Additionally, the GEPIA2 database was used to verify the expression levels of the 10 hub genes in tumor and normal tissues. As shown in Fig. 6a-j, the expression levels of the 10 hub genes were all statistically significant (*P* < 0.01) in OC and normal tissues on the basis of gene expression profiles from The Cancer Genome Atlas (TCGA) and the genotype-tissue expression (GTEx) projects. The results of the expression trends in OC patients and healthy people based on the GEPIA2 database were in accordance with those based on the GEO datasets (Fig. 6a-j and Table 2). Thus, both the GEPIA2 and GEO databases indicated that the mRNA expression levels of the 10 hub genes were upregulated in tumor tissues (Fig. 6a-j and Table 2). Additionally, the expression levels of the 10 hub genes in different stage OC patients are shown in Fig. 7. According to these results, it is easy to see that there were significant variations in the expression levels of BUB1B [Pr (>F) < 0.001] (Fig. 7b), FOXM1 [Pr (>F) = 0.007] (Fig. 7e), KIF4A [Pr (>F) = 0.022] (Fig. 7f), RRM2 [Pr (>F) = 0.025] (Fig. 7h), and TYMS [Pr (>F) = 0.002] (Fig. 7j) in OC patients in different stages. The overall trends indicated that the expression of the above five genes decreased gradually with the continuous progression of OC (Fig. 7).

### miRNA-hub gene network

**Fig. 8:**
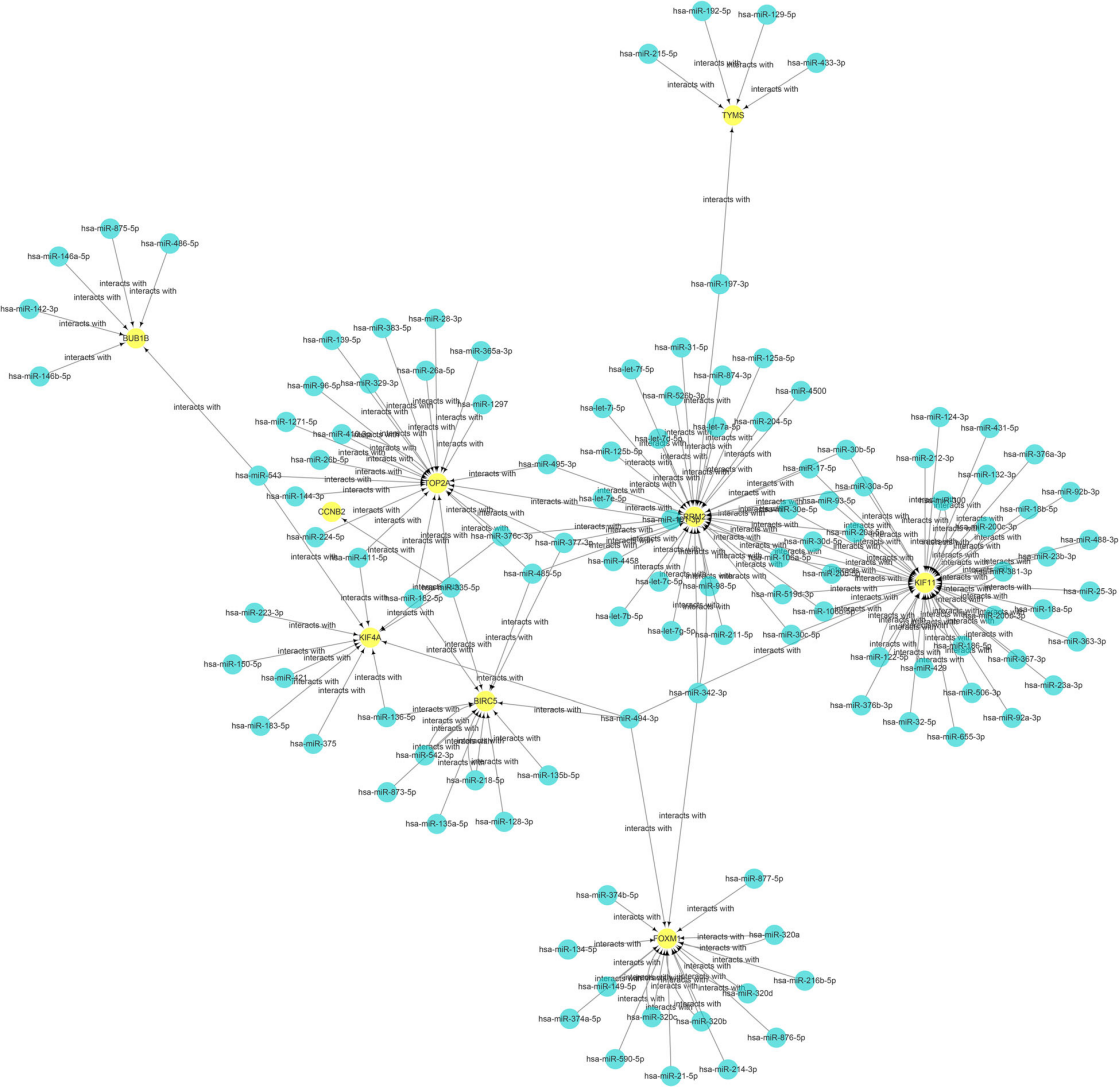
Interaction network between hub genes and targeted miRNAs. Hub genes are presented in yellow circles, whereas targeted miRNAs are shown in blue circles. The interaction between the hub genes and related miRNAs is shown in the form of arrows

ENCORI was applied to screen the targeted miRNAs of the hub genes. The miRNAs predicted by at least two databases (among the following databases: miRanda, PITA, PicTar, and TargetScan) were identified as the targeted miRNAs of the hub genes. Then, Cytoscape software was used to draw the miRNA-hub gene network. As illustrated in Fig. 8, the interaction network consists of 9 hub genes and 116 miRNAs. Moreover, the contribution level of the miRNAs to their surrounding hub genes is presented as the number of arrows (Fig. 8). According to the top 10 molecules in the network ranked by their degree of connectivity using cytoHubba, KIF11 (degree score = 47), RRM2 (degree score = 38), TOP2A (degree score = 23), and FOXM1 (degree score = 20) were clearly the four interactive hub genes that most miRNAs would target (Fig. 8), followed by BIRC5 (degree score = 14), KIF4A (degree score = 11), BUB1B (degree score = 6), and TYMS (degree score = 5). Furthermore, hsa-miR-377-3p (degree score = 4) and hsa-miR-335-5p (degree score = 4) were the top two interactive miRNAs that targeted the most target hub genes (Fig. 8). Additionally, the respective miRNAs targeting the 10 hub genes are presented in Additional file 14: Table S5.

### Drug-gene interaction

A total of 62 potential drugs for treating OC patients were identified when the drug-gene interactions were explored using DGIdb (Table 3). In this study, according to the statistically significant results of the survival analysis, TYMS and BIRC5 were selected as the potential targets of 62 drugs (Table 3). Both hub genes have been verified as current chemotherapy targets based on cBioPortal (Fig. 5a). As demonstrated in Table 3, the promising 62 candidate drugs were all approved by the FDA. Most potential drugs might interact with the TYMS (32/62), either in some unknown manners or as an inhibitor (Table 3). The downstream interaction networks of TYMS and BIRC5 were constructed by STITCH (Additional file 15: Figure S10). As shown in Additional file 15: Figure S10, TYMS might have downstream effects on 5-thymidylic acid (poly(dT)), 5-fluorouracil, deoxyuridine monophosphate (dUMP), pemetrexed, methotrexate, OSI-7904 L, raltitrexed, FdUMP, dihydrofolate, and dihydrofolate reductase (DHFR) (Additional file 15: Figure S10A), whereas BIRC5 might have downstream effects on exportin 1 (XPO1), poly-like kinase 1 (PLK1), cyclin-dependent kinase 1 (CDK1), cell division cycle associated 8 (CDCA8), aurora kinase A (AURKA), aurora kinase (AURKB), inner centromere protein antigens (INCENP), baculoviral IAP repeat-containing 2 (BIRC2), caspase 9 (CASP-9), and IAP-binding mitochondrial protein (DIABLO) (Additional file 15: Figure S10B).

**Table 3:** Candidate drugs targeted by the hub genes

No.	Gene	Drug	Interaction types	Sources	PMIDs
1	TYMS	ETOPOSIDE	–	PharmGKB	–
2	TYMS	IRINOTECAN	–	CIViC	20,628,391
3	TYMS	VINCRISTINE	–	NCI	2,804,079
4	TYMS	FOLIC ACID	–	PharmGKB	–
5	TYMS	TOPOTECAN	–	NCI	10,803,925
6	TYMS	CYTARABINE	–	PharmGKB	–
7	TYMS	MITOMYCIN	–	NCI	15,218,314
8	TYMS	PREDNISONE	–	PharmGKB	–
9	TYMS	TRIFLURIDINE	inhibitor	DrugBank	18,600,528; 16,902,987; 20,372,850; 19,816,940; 19,886,911; 4,719,131; 6,436,227; 15,571,283; 17,179,993; 14,719,072; 16,010,590; 15,125,867; 11,752,352; 18,798,063; 6,010,427
10	TYMS	DAUNORUBICIN	–	NCI	2,967,076
11	TYMS	HYDROCORTISONE	–	NCI	2,707,640
12	TYMS	PRALATREXATE	inhibitor	DrugBank	23,409,799
13	TYMS	PHENTOLAMINE	–	NCI	2,866,100
14	TYMS	TRIMETHOPRIM	inhibitor	DrugBank	8,538,681; 10,090,784; 10,592,235; 17,139,284; 8,920,005; 17,016,423; 19,622,858
15	TYMS	INDOMETHACIN	–	NCI	2,707,640
16	TYMS	GEMCITABINE	inhibitor	DrugBank	16,563,096; 15,795,320; 17,166,391; 16,818,276; 11,752,352; 15,655,942; 16,818,496
17	TYMS	ASPARAGINASE	–	PharmGKB	–
18	TYMS	CAPECITABINE	inhibitor	ClearityFoundationBiomarkers; ClearityFoundationClinicalTrial; GuideToPharmacologyInteractions; ChemblInteractions; DrugBank	15,134,221; 16,926,630; 15,866,500; 15,132,128; 11,752,352; 15,709,193
19	TYMS	FLOXURIDINE	inhibitor	TdgClinicalTrial; ClearityFoundationClinicalTrial; ChemblInteractions; TEND; DrugBank	10,697,523; 10,891,536; 10,697,524; 10,482,907; 10,553,409
20	TYMS	LEUCOVORIN	–	TdgClinicalTrial; TEND	–
21	TYMS	TAMOXIFEN	–	NCI	9,615,734
22	TYMS	VERAPAMIL	–	NCI	3,436,366
23	TYMS	RALTITREXED	inhibitor	PharmGKB; TdgClinicalTrial; GuideToPharmacologyInteractions; TEND; DrugBank	18,773,878; 10,598,555; 10,430,100; 10,592,235; 11,752,352; 10,499,608; 10,482,907; 10,047,461; 10,496,350
24	TYMS	PEMETREXED DISODIUM	inhibitor	ChemblInteractions	–
25	TYMS	DEXAMETHASONE	–	NCI	2,707,640; 3,398,844
26	TYMS	INTERFERON GAMA-1B	–	NCI	1,557,656
27	TYMS	FLUOROURACIL	inhibitor	ClearityFoundationClinicalTrial; ChemblInteractions; CIViC; DrugBank	16,563,096; 20,628,391; 16,609,021; 16,719,540; 16,596,248; 16,538,493; 11,752,352; 15,353,299
28	TYMS	OXALIPLATIN	–	PharmGKB	–
29	TYMS	TEGAFUR	–	TdgClinicalTrial	–
30	TYMS	SULFASALAZINE	–	PharmGKB	–
31	TYMS	PEMETREXED (CHEMBL1201258)	inhibitor	ClearityFoundationBiomarkers; TdgClinicalTrial; GuideToPharmacologyInteractions; CIViC; TEND	23,645,741; 23,060,591; 21,367,480; 26,502,926
32	TYMS	METHOTREXATE	–	CIViC	23,652,803
33	BIRC5	IRINOTECAN	–	NCI	15,956,246
34	BIRC5	INTERFERON BETA-1A	–	NCI	12,117,359
35	BIRC5	DOXORUBICIN	–	NCI	16,211,302
36	BIRC5	CYTARABINE	–	NCI	14,713,572
37	BIRC5	TRASTUZUMAB	–	NCI|CIViC	16,452,223; 23,204,226
38	BIRC5	LAPATINIB	–	NCI	16,452,223
39	BIRC5	PROXYPHYLLINE	–	NCI	12,895,357
40	BIRC5	PACLITAXEL	–	ClearityFoundationBiomarkers; NCI	16,211,241; 15,347,474
41	BIRC5	DOCETAXEL	–	ClearityFoundationBiomarkers	–
42	BIRC5	FLUTAMIDE	–	NCI	15,735,703
43	BIRC5	VORINOSTAT	–	NCI	16,951,239
44	BIRC5	PRASTERONE	–	NCI	16,461,558
45	BIRC5	ARSENIC TRIOXIDE	–	NCI	15,587,394; 16,950,207
46	BIRC5	ERLOTINIB	–	NCI	17,047,074
47	BIRC5	INDOMETHACIN	–	NCI	17,270,149
48	BIRC5	IMATINIB	–	NCI	16,254,145
49	BIRC5	ROMIDEPSIN	–	NCI	14,767,553
50	BIRC5	EPIRUBICIN	–	NCI	16,608,080
51	BIRC5	PLICAMYCIN	–	NCI	17,124,180
52	BIRC5	OMACETAXINE MEPESUCCINATE	–	NCI	15,854,289
53	BIRC5	MYCOPHENOLIC ACID	–	NCI	15,571,295
54	BIRC5	DEXAMETHASONE	–	NCI	16,787,583
55	BIRC5	FLUOROURACIL	–	NCI	15,067,352
56	BIRC5	DACTINOMYCIN	–	NCI	15,883,285
57	BIRC5	CALCITONIN	–	NCI	16,222,118
58	BIRC5	CARBOPLATIN	–	NCI	15,347,474
59	BIRC5	EPOETIN ALFA	–	NCI	17,112,829
60	BIRC5	TRETINOIN	–	NCI	11,939,262; 14,587,026; 16,403,261
61	BIRC5	METHOTREXATE	–	NCI	15,670,151
62	BIRC5	SULINDAC	–	NCI	16,707,021; 16,950,207

These data were obtained from the DGIdb database

## Discussion

Although great progress on surgical and medical therapy has been made for OC, the overall mortality of OC remains the fifth cause of death among malignant gynecologic tumors. The causes of death from OC are mainly attributed to a lack of detection methods at an early stage, a high tendency for metastasis, and chemotherapy resistance [12, 13]. Therefore, exploring reliable biomarkers and precise molecular mechanisms for the early diagnosis, treatment, and prognosis of OC is urgent and necessary. In recent years, with the rapid development of bioinformatics, an increasing amount of microarray and sequencing data have provided a convenient and comprehensive platform to explore general genetic alterations, identify DEGs, and elucidate molecular mechanisms for the diagnosis, therapy, and prognosis of tumors [14].

In this study, four GEO datasets, namely, GSE54388, GSE69428, GSE36668, and GSE40595, were selected to screen DEGs. Applying the GEO2R tool and FunRich software, the intersection of 171 DEGs was identified, including 114 upregulated DEGs and 57 downregulated DEGs. Then, GO and KEGG pathway analyses of the DEGs were performed by DAVID. The KEGG pathway results showed that the upregulated DEGs were mainly associated with metabolic pathways, the biosynthesis of antibiotics, the biosynthesis of amino acids, the cell cycle, and HTLV-I infection, whereas the downregulated DEGs were not enriched. These results also provided significant clues to studying molecular interactions in the progression of OC. Indeed, many studies have indicated that metabolic pathways and the cell cycle are highly associated with the tumorigenesis and progression of OC [15–17]. For example, autophagy is well known to affect various processes, such as survival under harsh metabolic conditions, and the actual level of autophagy in OC cells could also be affected by cancer-associated fibroblasts [15]. Previously, many studies have reported that antibiotics, such as minocycline, salinomycin, monensin, and pegylated liposomal doxorubicin, could be effective in treating OC [18–21]. Furthermore, Plewa S et al. once quantitated 42 serum-free amino acids and distinguished amino acid metabolic pathways related to OC growth and development [22]. However, the association between HTLV-I and OC remains unclear. In this study, the upregulated DEGs were mainly involved in biological functions such as cell division, the nucleus, and protein binding. The downregulated DEGs were mainly enriched in biological functions such as the negative regulation of transcription from the RNA polymerase II promoter, the protein complex, the apicolateral plasma membrane, and glycosaminoglycan binding.

Furthermore, a PPI network with 168 nodes and 450 edges was constructed based on the DEGs, and 10 hub genes with the highest degrees of connectivity in the PPI network were identified. Subsequently, survival analysis of these hub genes demonstrated that 8 upregulated hub genes (CCNB2, TYMS, KIF11, KIF4A, BIRC5, BUB1B, FOXM1, and CDC20) were significantly associated with a poorer PFS in OC patients at an early stage (stages 1 and 2). Then, the GEPIA2 database was used to further verify the expression levels of these hub genes in tumor and normal tissues, and the results were in accordance with those from the GEO database. The expression levels of hub genes in different stage OC patients were also conducted. Considering the significant variations in the expression levels of KIF4A and TYMS in early and advanced stage OC patients (Fig. 7), we hypothesized that this result may explain why KIF4A and TYMS were related to poorer PFS in early stage (stages 1 and 2) OC and SOC patients (Fig. 4g, k, Additional file 9: Figure S5G, & K), whereas the 2 hub genes described above were associated with better PFS in advanced stage (stages 3 and 4) OC and SOC patients (Additional file 12: Figure S8 and Additional file 13: Figure S9). Thus, we further hypothesized whether KIF4A and TYMS might be considered as the tumor marker in the diagnosis of early OC. It is well known that tumor invasion and metastasis are always accompanied by complicated molecular regulatory mechanisms. In this study, the expression levels of all hub genes were dysregulated in OC tissues compared with normal tissues, meaning that these genes may be crucial to tumorigenesis and progression in OC. Although many studies have studied these hub genes connected with the diagnosis, treatment, and prognosis of various carcinomas, the molecular mechanisms underlying the tumorigenesis and progression of OC have not been fully understood.

First, kinesin family member 4A (KIF4A) is a microtubule-based motor protein that has been reported to be associated with various cancers, such as oral squamous cell carcinoma, cervical cancer, breast cancer, prostate cancer, colorectal cancer, and lung cancer [23–28]. However, there have been no studies conducted on KIF4A in regard to OC. In this study, the downregulated expression of KIF4A was correlated with poorer PFS in OC patients, especially in SOC patients at an early stage (Fig. 4g, Additional file 6: Figure S4G). We also found 11 potential miRNAs (hsa-miR-183-5p, hsa-miR-223-3p, hsa-miR-136-5p, hsa-miR-150-5p, hsa-miR-376c-3p, hsa-miR-375, hsa-miR-335-5p, hsa-miR-494-3p, hsa-miR-411-5p, hsa-miR-421, and hsa-miR-543) that KIF4A might target (Fig. 8).

The overexpression of many cyclins has been reported in many tumors [29]; cyclin B2 (CCNB2) is located on the Golgi apparatus during the whole cell cycle and may cause a compromised G2/M transition in Hec1-depleted mouse oocytes [30, 31]. However, the association between CCNB2 and OC is not clearly understood. Cell division cycle 20 (CDC20) is an activator of the ligase anaphase-promoting complex/C (APC/C) that has been identified as a key candidate gene in OC patients based on many bioinformatics studies [32, 33]. High expression levels of CDC20 have been reported to be correlated with poor prognosis in a variety of cancers [34–36]. However, its specific molecular mechanisms underlying OC have not been fully explained. In this study, the survival analysis of CDC20 in OC patients at an early stage suggested that higher CDC20 expression could be related to poorer prognosis (Fig. 4e).

Thymidylate synthase (TYMS) is crucial for DNA synthesis in both normal and tumor cells. Biason P et al. calculated six TYMS polymorphisms related to OS in epithelial OC (EOC) through a multivariate analysis and found that the TYMS 1494ins/del, 1053C > T and IVS6-68C > T polymorphisms might be considered prognostic markers for OS in EOC patients [37]. Additionally, Kelemen LE et al. also reported that re495139 in the TYMS-ENOSF1 region was associated with the risk of OC by evaluating large samples of assembled cases [38, 39]. KIF11, a member of the kinesin-like protein family, was once identified as a hub gene in OC by Zhanzhan Xu et al. [8], and they also found that KIF11 was potentially targeted by hsa-miR-424 and hsa-miR-381. A recent study explored whether death receptor 6 can promote OC cell migration by interacting with KIF11 and TNF receptor-associated factor 4 [40]. In this study, KIF11 was also considered a chemotherapy target for OC using cBioPortal (Fig. 5a). Ribonucleotide reductase M2 (RRM2) is the regulatory subunit of ribonucleotide reductase and is not only involved in the tumorigenesis and progression of a variety of carcinomas (such as colon, breast, and pancreatic cancer) [41–43] but also leads to resistance to cancer chemotherapy [44]. Katherine M Aird et al. found that the knockdown of RRM2 expression could inhibit the growth of human EOC cells by triggering cellular senescence [45], which suggests that RRM2 might be a novel prosenescence therapeutic strategy to improve the OS of EOC patients. Our drug-hub gene interaction result was consistent with that of Katherine M Aird et al. Type 2 topoisomerase alpha (TOP2A) is a gene close to the HER2 gene that encodes a nuclear enzyme that is crucial in the cell cycle. Mano et al. first explored TOP2A amplifications in EOC [46], whereas in our study, we found that the type of alteration for TOP2A in SOC mainly included truncating mutations, missense mutations, and deep deletions (Fig. 5b). Mitotic checkpoint serine/threonine kinase B (BUB1B), the mammalian homolog of yeast Mad 3, is susceptible to cancer and causes chromosome loss and apoptotic cell death in human cancer cells [47, 48]. Many studies have reported that BUB1B is highly associated with advanced stages, serous histology and high grades in EOC, and a weakened spindle checkpoint with the downregulated expression of BUB1B is also related to acquired paclitaxel resistance in OC cells [49, 50]. Forkhead box protein M1 (FOXM1), a transcription factor with a “winged helix” DNA-binding domain, plays a significant role in tumor progression through cell proliferation, tumor invasion, migration, and angiogenesis [51, 52]. Yu Wang et al. reported that FOXM1 promotes the reprogramming of glucose metabolism in EOC by activating GLUT1 and HK2 transcription [53]. Finally, baculoviral inhibitor of apoptosis repeat-containing 5 (BIRC5) is an inhibitor of apoptosis that is always absent in normal tissues and is associated with various cancers, such as breast cancer and OC [54, 55]. Epithelial to mesenchymal transition (EMT) is a biological process that is associated with tumor metastasis and chemoresistance in ovarian tumors [56]. Zhao G et al. reported that higher expression of BIRC5 always promoted EMT in ovarian cancer cells [57]. In this study, both the cBioPortal and DGIdb databases considered BIRC5 to be a target of chemotherapy drugs used to treat OC (Fig. 5a, Table 3).

Furthermore, 10 genes were identified as the hub genes, and their expression levels in OC patients of different stages were all verified according to both the GEO and GEPIA2 databases. A list of 62 drugs with potential therapeutic efficacy against OC were selected (Table 3). For the 10 hub genes, the potential gene targets of these drugs were TYMS and BIRC5. However, the mechanisms of most of these drugs were unknown. The effects of the screened drugs were verified for many other types of cancers such as lung cancer, liver cancer, and colon cancer. Only a few of the TYMS- and BIRC5-targeting drugs have been used for OC treatment, such as fluorouracil/oxaliplatin (5-fluorouracil can induce the upregulation of human TYMS expression through TYMS mRNA transcription) [58], paclitaxel (the inhibition of BIRC5 expression can activate cell responses to paclitaxel treatment) [57], and carboplatin [59]. Additional studies and clinical trials are needed to identify and explore the drugs that are effective for OC treatment in future. Nevertheless, this study used multiple available databases to provide novel insights into the OC pathogenesis and treatment, and the identified conventional drugs might find potential new uses.

Therefore, considering the crucial roles of these 10 hub genes on the basis of this study and other previous studies mentioned above, further studies may be focused on exploring their precise mechanisms in the tumorigenesis and prognosis of OC, especially studying KIF4A, CCNB2, and CDC20. There were still several limitations in this study. One is that the microarray data and other analyses were obtained from many public databases, not generated by authors. The other is that only the TCGA and GEPIA2 databases were used to verify the expression levels of the hub genes, and experiments were not performed by the authors. Furthermore, due to a lack of experimental studies and verifications, we could not further explore how hub gene-miRNAs networks have effects on the diagnosis and therapy of ovarian cancer in depth. Despite these limitations, this study may provide more accurate results based on integrated bioinformatic analysis compared to the single dataset studies.

## Conclusions

In summary, a total of 171 DEGs, including 114 upregulated DEGs and 57 downregulated DEGs in OC, were screened through integrated bioinformatic analysis, and 10 hub genes, namely, KIF4A, CDC20, CCNB2, TOP2A, RRM2, TYMS, KIF11, BIRC5, BUB1B, and FOXM1, may play crucial roles in the tumorigenesis and prognosis of OC. Among these hub genes, one gene, KIF4A, has not been previously reported to be related to OC, which indicates that KIF4A might be a potential biomarker for OC diagnosis and prognosis at an early stage. Additionally, the miRNA-hub gene network and potential targeted drugs related to these hub genes were constructed and selected, respectively, and may contribute to studying the mechanisms of OC. In-depth molecular mechanisms of the novel hub genes in OC are needed in future, and relevant experimental models can be constructed on the basis of these genes for the early detection, prognostic judgment, risk assessment, and targeted therapy of OC.

## Supplementary information

**Additional file 1:** Heatmap of the differentially expressed genes (DEGs) between OC tissue and normal tissues.

**Additional file 2:** GO analysis of the downregulated DEGs.

**Additional file 3:** Upregulated genes identified in each category for the functional enrichment analysis.

**Additional file 4:** PPI network of 168 DEGs.

**Additional file 5:** Interaction network between the DEGs and their related genes.

**Additional file 6:** The four significant modules selected from the PPI network.

**Additional file 7:** KEGG pathway analysis of each module.

**Additional file 8:** Top 10 hub genes in the PPI network ranked by the degree method.

**Additional file 9:** Progression free survival analyses of the hub genes in early stage (stages 1 and 2) SOC patients.

**Additional file 10:** Progression free survival analyses of the hub genes in all stage OC patients.

**Additional file 11:** Progression free survival analyses of the hub genes in all stage SOC patients.

**Additional file 12:** Progression free survival analyses of the hub genes in advanced stage (stages 3 and 4) OC patients.

**Additional file 13:** Progression free survival analyses of the hub genes in advanced stage (stages 3 and 4) SOC patients.

**Additional file 14:** The respective miRNAs targeting the 10 hub genes.

**Additional file 15:** Targetable TYMS and BIRC5 subnetwork.

## Data Availability

The datasets used and/or analyzed during the current study are available from the corresponding author upon reasonable request.

## Abbreviations

BP: Biological process
CC: Cellular component
DAVID: Database for Annotation, Visualization and Integrated Discovery
DEGs: Differentially expressed genes
DGIdb: Drug-Gene Interaction Database
ENCORI: The Encyclopedia of RNA Interactomes
FDA: Food and Drug Administration
GEO: Gene Expression Omnibus
GEPIA2: Gene Expression Profiling Interactive Analysis
GO: Gene Ontology
KEGG: Kyoto Encyclopedia of Genes and Genomes
MCODE: Molecular Complex Detection
MF: Molecular function
OC: Ovarian cancer
PFS: Progression free survival
PPI: Protein-protein interaction
SOC: Serous ovarian cancer
STRING: Search Tool for the Retrieval of Interacting Genes/Proteins
TCGA: The Cancer Genome Atlas
