# Supplementary material for: Integrated bioinformatics analysis for the screening of hub genes and therapeutic drugs in ovarian cancer
Source: J Ovarian Res. 2020 Jan 27;13:10. doi: 10.1186/s13048-020-0613-2 (PMC6986075; doi:10.1186/s13048-020-0613-2)
Supplement: Supplementary file 1 — Additional file 1: Heatmap of the differentially expressed genes (DEGs) between OC tissue and normal tissues. [file 13048_2020_613_MOESM1_ESM.docx]

**Additional file 1.**

**Figure S1. Heatmap of the differentially expressed genes (DEGs) between OC tissue and normal tissues.**

**
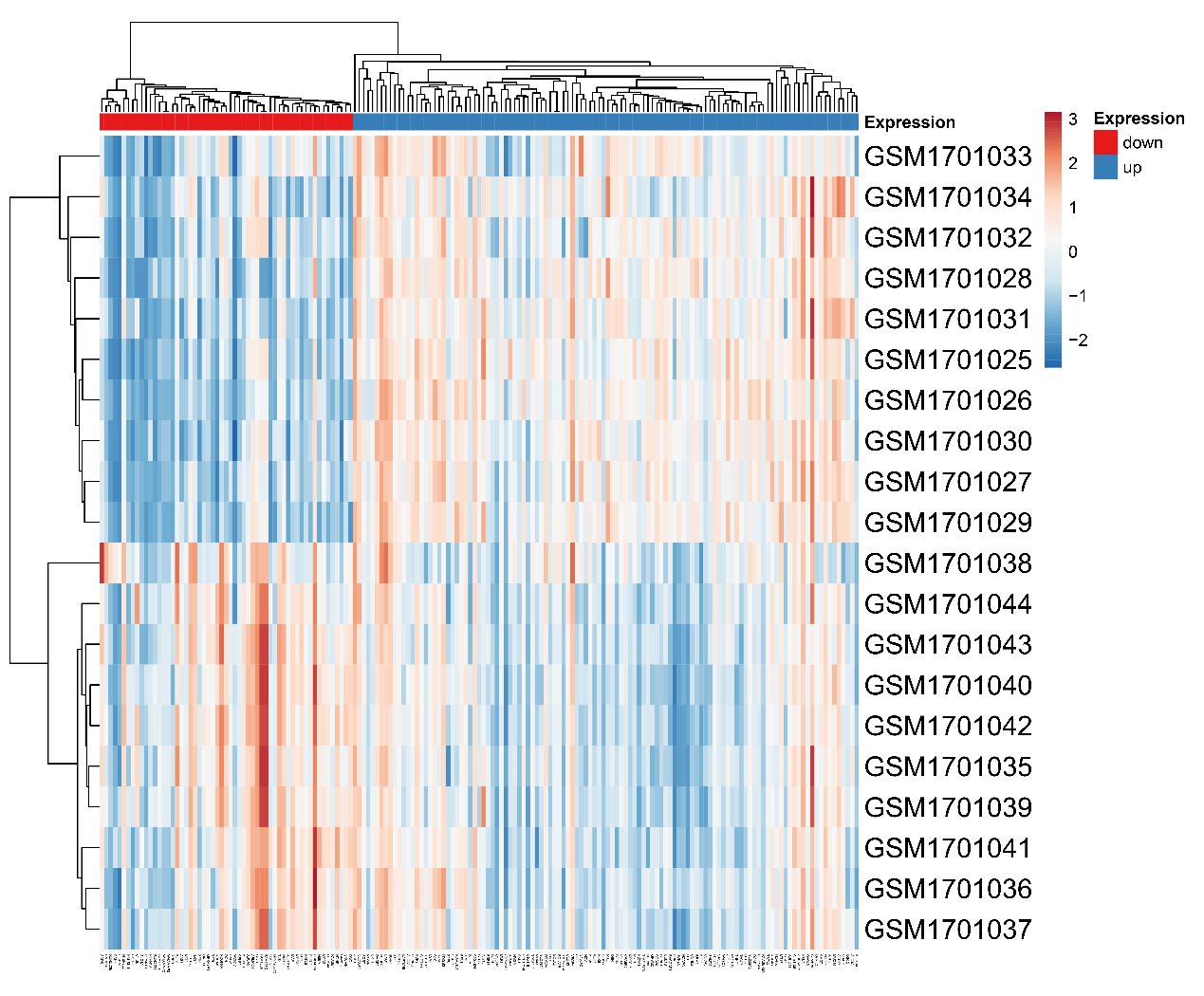
**
