# Supplementary material for: Integrated bioinformatics analysis for the screening of hub genes and therapeutic drugs in ovarian cancer
Source: J Ovarian Res. 2020 Jan 27;13:10. doi: 10.1186/s13048-020-0613-2 (PMC6986075; doi:10.1186/s13048-020-0613-2)
Supplement: Supplementary file 2 — Additional file 2: GO analysis of the downregulated DEGs. [file 13048_2020_613_MOESM2_ESM.docx]

**Additional file 2.**

**Table S1. GO analysis of the downregulated DEGs**

| Category | Term | Count | P-value | Genes |
| --- | --- | --- | --- | --- |
| GOTERM_BP_DIRECT | GO:0000122~negative regulation of transcription from RNA polymerase II promoter | 8 | 0.002 | MAF, MEF2C, N4BP2L2, ZBTB20, MITF, TLE4, ZEB2, FOXP2 |
| GOTERM_BP_DIRECT | GO:0045944~positive regulation of transcription from RNA polymerase II promoter | 7 | 0.038 | MAF, MEF2C, KAT2B, MITF, ZEB2, DCN, KLF2 |
| GOTERM_BP_DIRECT | GO:0007519~skeletal muscle tissue development | 4 | <0.001 | MEF2C, DMD, DCN, FOXP2 |
| GOTERM_BP_DIRECT | GO:0016032~viral process | 4 | 0.041 | KAT2B, MPDZ, IL6ST, PDGFRA |
| GOTERM_BP_DIRECT | GO:0014066~regulation of phosphatidylinositol 3-kinase signaling | 3 | 0.017 | PDGFRA, PIP5K1B, PTPN13 |
| GOTERM_CC_DIRECT | GO:0043234~protein complex | 5 | 0.023 | MEF2C, DMD, MITF, PRKAR1A, PDGFRA |
| GOTERM_CC_DIRECT | GO:0016327~apicolateral plasma membrane | 2 | 0.044 | THBD, MPDZ |
| GOTERM_MF_DIRECT | GO:0005539~glycosaminoglycan binding | 2 | 0.049 | DCN, NDNF |
