# Supplementary material for: Integrated bioinformatics analysis for the screening of hub genes and therapeutic drugs in ovarian cancer
Source: J Ovarian Res. 2020 Jan 27;13:10. doi: 10.1186/s13048-020-0613-2 (PMC6986075; doi:10.1186/s13048-020-0613-2)
Supplement: Supplementary file 3 — Additional file 3: Upregulated genes identified in each category for the functional enrichment analysis. [file 13048_2020_613_MOESM3_ESM.docx]

**Additional file 3.**

**Table S2. Upregulated genes identified in each category for the functional enrichment analysis**

| Category | Term | Count | 1. value | Genes |
| --- | --- | --- | --- | --- |
| GOTERM_BP_DIRECT | GO:0051301~cell division | 13 | <0.001 | KIF11, CDC20, NDC80, BIRC5, PTTG1, TACC3, CCNE1, CCNB2, TIMELESS, SAC3D1, NCAPG, BUB1B, CDCA3 |
| GOTERM_BP_DIRECT | GO:0007067~mitotic nuclear division | 11 | <0.001 | KIF11, CCNB2, TIMELESS, SAC3D1, BUB1B, BIRC5, NDC80, CDC20, PBK, PTTG1, CDCA3 |
| GOTERM_BP_DIRECT | GO:0051726~regulation of cell cycle | 7 | <0.001 | CCNE1, BAK1, CCNB2, OVOL2, DTL, FOXM1, KIAA0101 |
| GOTERM_BP_DIRECT | GO:0006260~DNA replication | 7 | <0.001 | TIMELESS, DTL, RRM2, KIAA0101, RNASEH2A, MCM4, FEN1 |
| GOTERM_BP_DIRECT | GO:0006890~retrograde vesicle-mediated transport, Golgi to ER | 5 | 0.002 | KIF4A, KIF11, ARF3, RACGAP1, COPE |
| GOTERM_BP_DIRECT | GO:0006268~DNA unwinding involved in DNA replication | 3 | 0.002 | HMGA1, MCM4, TOP2A |
| GOTERM_BP_DIRECT | GO:0006974~cellular response to DNA damage stimulus | 7 | 0.002 | DDX39A, TIMELESS, DTL, KIAA0101, H2AFX, DYRK2, TOP2A |
| GOTERM_BP_DIRECT | GO:0000083~regulation of transcription involved in G1/S transition of mitotic cell cycle | 3 | 0.009 | CCNE1, TYMS, RRM2 |
| GOTERM_BP_DIRECT | GO:0007052~mitotic spindle organization | 3 | 0.014 | KIF11, GPSM2, NDC80 |
| GOTERM_BP_DIRECT | GO:0010046~response to mycotoxin | 2 | 0.018 | BAK1, ASS1 |
| GOTERM_CC_DIRECT | GO:0005654~nucleoplasm | 33 | <0.001 | DPP3, KIF4A, AURKAIP1, FOXM1, CRABP2, KIAA0101, SCRIB, CCNE1, TYMS, ISG15, NR2F6, LSM4, H2AFX, DYRK2, TOP2A, FEN1, KLK6, DDX39A, DTL, FDPS, WHSC1, BIRC5, CDC20, RACGAP1, RNASEH2A, HMGA1, MCM4, CCNB2, TIMELESS, RRM2, ESRP1, PUF60, COPE |
| GOTERM_CC_DIRECT | GO:0005829~cytosol | 37 | <0.001 | KIF4A, ASS1, CRABP2, CTPS1, PTTG1, ATP6V1B1, CCNE1, TYMS, BAK1, TPI1, APOA1, ISG15, NCAPG, SLC2A1, IDH2, LSM4, TSTA3, PIK3R3, CDCA3, DHCR24, ENO1, IRAK1, KIF11, FDPS, NDC80, BIRC5, CDC20, RACGAP1, HMGA1, TNNT1, CCNB2, RRM2, KLHL14, SORT1, BUB1B, PSAT1, COPE |
| GOTERM_CC_DIRECT | GO:0070062~extracellular exosome | 28 | 0.006 | DPP3, PXDN, ASS1, CLDN3, NDUFB9, CRABP2, BOLA2B, ATP6V1B1, SCRIB, CDH6, CD47, TPI1, APOA1, SLC2A1, IDH2, AIF1L, H2AFX, TSTA3, SLC39A4, ENO1, SHMT2, BOLA2, RACGAP1, ARF3, DSC2, SLC38A1, PSAT1, MUC16 |
| GOTERM_CC_DIRECT | GO:0005737~cytoplasm | 44 | 0.008 | DPP3, KIF4A, HMGB3, ASS1, FOXM1, CRABP2, KIAA0101, PTTG1, TPD52, ATP6V1B1, MLF2, SCRIB, NOV, TYMS, SAC3D1, NCAPG, AIF1L, GPSM2, DYRK2, TSTA3, TOP2A, PPP1R14B, MT1F, ENO1, DDX39A, KLK6, IRAK1, SHMT2, KLK8, KIF11, DTL, FDPS, WHSC1, BIRC5, CDC20, RACGAP1, TACC3, RNASEH2A, RRM2, VEGFA, BUB1B, PSAT1, ARL4C, DBN1 |
| GOTERM_CC_DIRECT | GO:0015630~microtubule cytoskeleton | 5 | 0.009 | KLK6, SHMT2, CCNB2, TIMELESS, TACC3 |
| GOTERM_CC_DIRECT | GO:0005634~nucleus | 45 | 0.009 | HMGB3, AURKAIP1, ASS1, FOXM1, E2F8, CRABP2, CYC1, KIAA0101, PTTG1, MLF2, CCNE1, TYMS, TPI1, CHD7, APOA1, OVOL2, NCAPG, NR2F6, H2AFX, DYRK2, TOP2A, FEN1, MT1F, ENO1, DHCR24, DDX39A, IRAK1, SHMT2, DTL, NDC80, HN1, WHSC1, BIRC5, CDC20, PBK, RACGAP1, MCAM, HMGA1, MYCL, MCM4, CCNB2, TIMELESS, RRM2, ESRP1, ARL4C |
| GOTERM_CC_DIRECT | GO:0005743~mitochondrial inner membrane | 8 | 0.016 | TYMS, SHMT2, MRPS15, MRPL12, AURKAIP1, NDUFB9, CYC1, IDH2 |
| GOTERM_CC_DIRECT | GO:0016323~basolateral plasma membrane | 5 | 0.022 | SLC4A11, SLC2A1, ATP6V1B1, SLC19A1, SCRIB |
| GOTERM_CC_DIRECT | GO:0005694~chromosome | 4 | 0.025 | KIF4A, HMGB3, DTL, WHSC1 |
| GOTERM_CC_DIRECT | GO:0005783~endoplasmic reticulum | 11 | 0.025 | KLK6, BAK1, PXDN, STC2, ASS1, LPCAT1, KLHL14, CRABP2, MAL, TPD52, DHCR24 |
| GOTERM_MF_DIRECT | GO:0005515~protein binding | 76 | <0.001 | DPP3, AURKAIP1, E2F8, CRABP2, PTTG1, TPD52, SLC52A2, NOV, CD47, BAK1, CCNE1, APOA1, ISG15, SLC2A1, NR2F6, LSM4, H2AFX, TOP2A, CDCA3, S100A2, DDX39A, IRAK1, DTL, PBK, TACC3, MCM4, HMGA1, TNNT1, DGAT1, TIMELESS, RRM2, VEGFA, ESRP1, BUB1B, SORT1, SLC38A1, DBN1, ARL4C, COPE, KIF4A, HMGB3, ASS1, NDUFB9, FOXM1, KIAA0101, MLF2, SCRIB, TPI1, CHD7, MRPL12, SAC3D1, NCAPG, GPSM2, TBC1D7, DYRK2, PIK3R3, FEN1, ENO1, MT1F, KLK6, LAPTM4B, C9ORF16, SHMT2, KLK8, COL4A1, NDC80, CDC20, BIRC5, MAL, WHSC1, RACGAP1, TMPRSS4, CCNB2, DSC2, PUF60, MUC16 |
| GOTERM_MF_DIRECT | GO:0042802~identical protein binding | 14 | 0.001 | DDX39A, BAK1, SHMT2, APOA1, ASS1, CLDN3, VEGFA, SLC2A1, GPSM2, CTPS1, BIRC5, NDC80, PUF60, S100A2 |
| GOTERM_MF_DIRECT | GO:0019899~enzyme binding | 9 | 0.001 | APOA1, STC2, SORT1, H2AFX, BIRC5, CDC20, HMGA1, TOP2A, DHCR24 |
| GOTERM_MF_DIRECT | GO:0046982~protein heterodimerization activity | 8 | 0.025 | IRAK1, BAK1, TIMELESS, VEGFA, H2AFX, BIRC5, TPD52, TOP2A |
| GOTERM_MF_DIRECT | GO:0003682~chromatin binding | 7 | 0.034 | SHMT2, CHD7, OVOL2, KIAA0101, WHSC1, HMGA1, TOP2A |
| GOTERM_MF_DIRECT | GO:0042803~protein homodimerization activity | 10 | 0.036 | IRAK1, BAK1, TYMS, STC2, TIMELESS, E2F8, VEGFA, BIRC5, TPD52, TOP2A |
| GOTERM_MF_DIRECT | GO:0000287~magnesium ion binding | 5 | 0.037 | IDH2, DYRK2, TOP2A, FEN1, ENO1 |
| GOTERM_MF_DIRECT | GO:0005542~folic acid binding | 2 | 0.077 | TYMS, SLC19A1 |
| GOTERM_MF_DIRECT | GO:0004523~RNA-DNA hybrid ribonuclease activity | 2 | 0.089 | RNASEH2A, FEN1 |
| GOTERM_MF_DIRECT | GO:0048037~cofactor binding | 2 | 0.094 | TYMS, BIRC5 |
| KEGG_PATHWAY | hsa01230:Biosynthesis of amino acids | 6 | <0.001 | SHMT2, TPI1, ASS1, IDH2, PSAT1, ENO1 |
| KEGG_PATHWAY | hsa04110:Cell cycle | 6 | 0.003 | CCNE1, CCNB2, BUB1B, CDC20, PTTG1, MCM4 |
| KEGG_PATHWAY | hsa01130:Biosynthesis of antibiotics | 7 | 0.005 | SHMT2, TPI1, ASS1, FDPS, IDH2, PSAT1, ENO1 |
| KEGG_PATHWAY | hsa01200:Carbon metabolism | 5 | 0.011 | SHMT2, TPI1, IDH2, PSAT1, ENO1 |
| KEGG_PATHWAY | hsa01100:Metabolic pathways | 17 | 0.018 | SHMT2, ASS1, NDUFB9, CYC1, FDPS, CTPS1, ATP6V1B1, TYMS, TPI1, DGAT1, LPCAT1, RRM2, IDH2, TSTA3, PSAT1, ENO1, DHCR24 |
| KEGG_PATHWAY | hsa03030:DNA replication | 3 | 0.031 | RNASEH2A, MCM4, FEN1 |
| KEGG_PATHWAY | hsa04066:HIF-1 signaling pathway | 4 | 0.037 | VEGFA, SLC2A1, PIK3R3, ENO1 |
| KEGG_PATHWAY | hsa05166:HTLV-I infection | 6 | 0.045 | SLC2A1, FDPS, BUB1B, CDC20, PTTG1, PIK3R3 |
| KEGG_PATHWAY | hsa04114:Oocyte meiosis | 4 | 0.054 | CCNE1, CCNB2, CDC20, PTTG1 |
| KEGG_PATHWAY | hsa05203:Viral carcinogenesis | 5 | 0.072 | CCNE1, BAK1, CDC20, PIK3R3, SCRIB |
