# Supplementary figures and images for: Integrated bioinformatics analysis for the screening of hub genes and therapeutic drugs in ovarian cancer
Source: J Ovarian Res. 2020 Jan 27;13:10. doi: 10.1186/s13048-020-0613-2 (PMC6986075; doi:10.1186/s13048-020-0613-2)

**Additional file 4.**

**Figure S2. PPI network of 168 DEGs.**

**
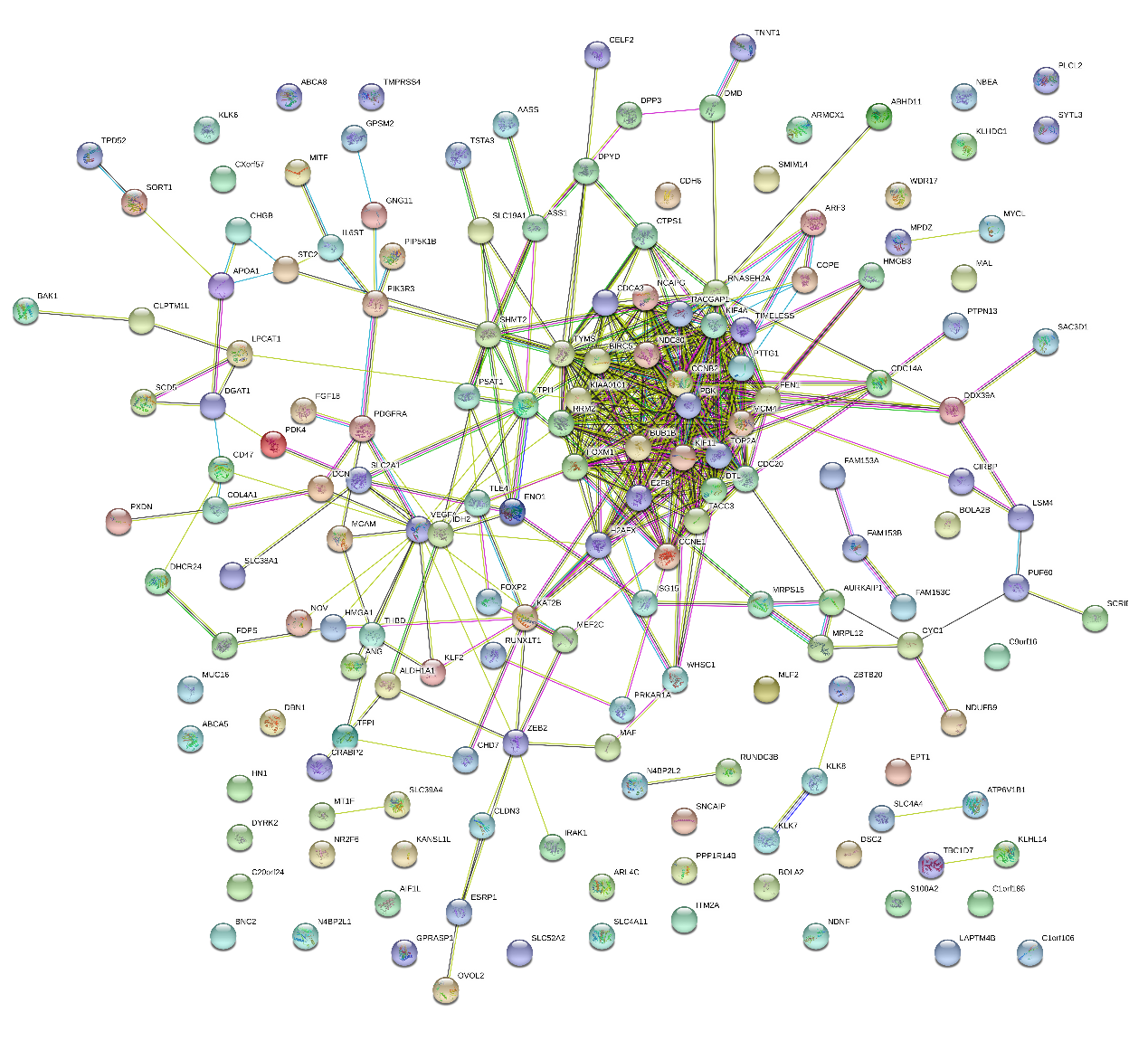
**

Supplement: Supplementary file 4 — Additional file 4: PPI network of 168 DEGs. [file 13048_2020_613_MOESM4_ESM.docx]

**Additional file 5.**

**Figure S3. Interaction network between the DEGs and their related genes.**

**
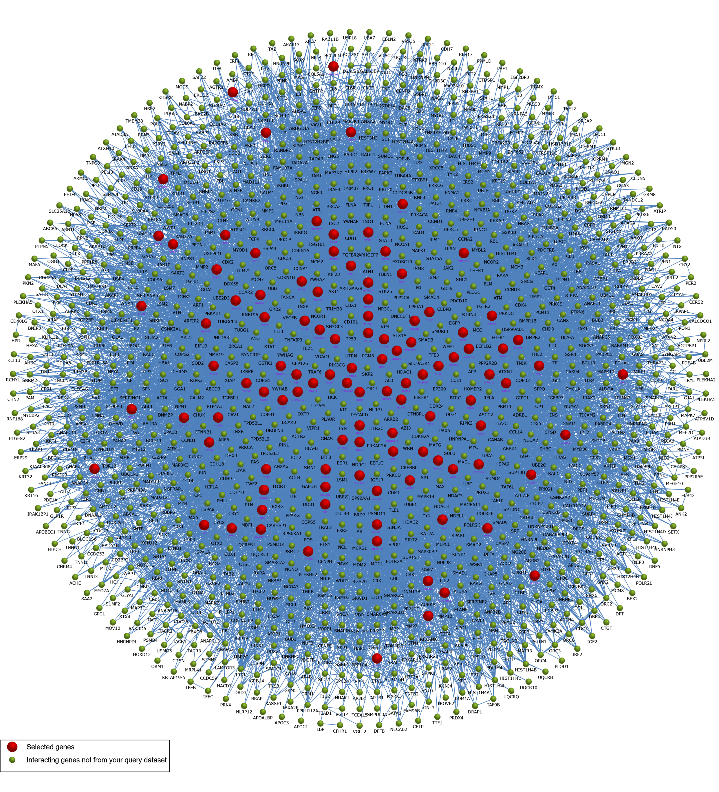
**

Supplement: Supplementary file 5 — Additional file 5: Interaction network between the DEGs and their related genes. [file 13048_2020_613_MOESM5_ESM.docx]
