# Supplementary material for: Integrated bioinformatics analysis for the screening of hub genes and therapeutic drugs in ovarian cancer
Source: J Ovarian Res. 2020 Jan 27;13:10. doi: 10.1186/s13048-020-0613-2 (PMC6986075; doi:10.1186/s13048-020-0613-2)
Supplement: Supplementary file 6 — Additional file 6: The four significant modules selected from the PPI network. [file 13048_2020_613_MOESM6_ESM.docx]

**Additional file 6.**

**Figure S4. The four significant modules selected from the PPI network.**

**
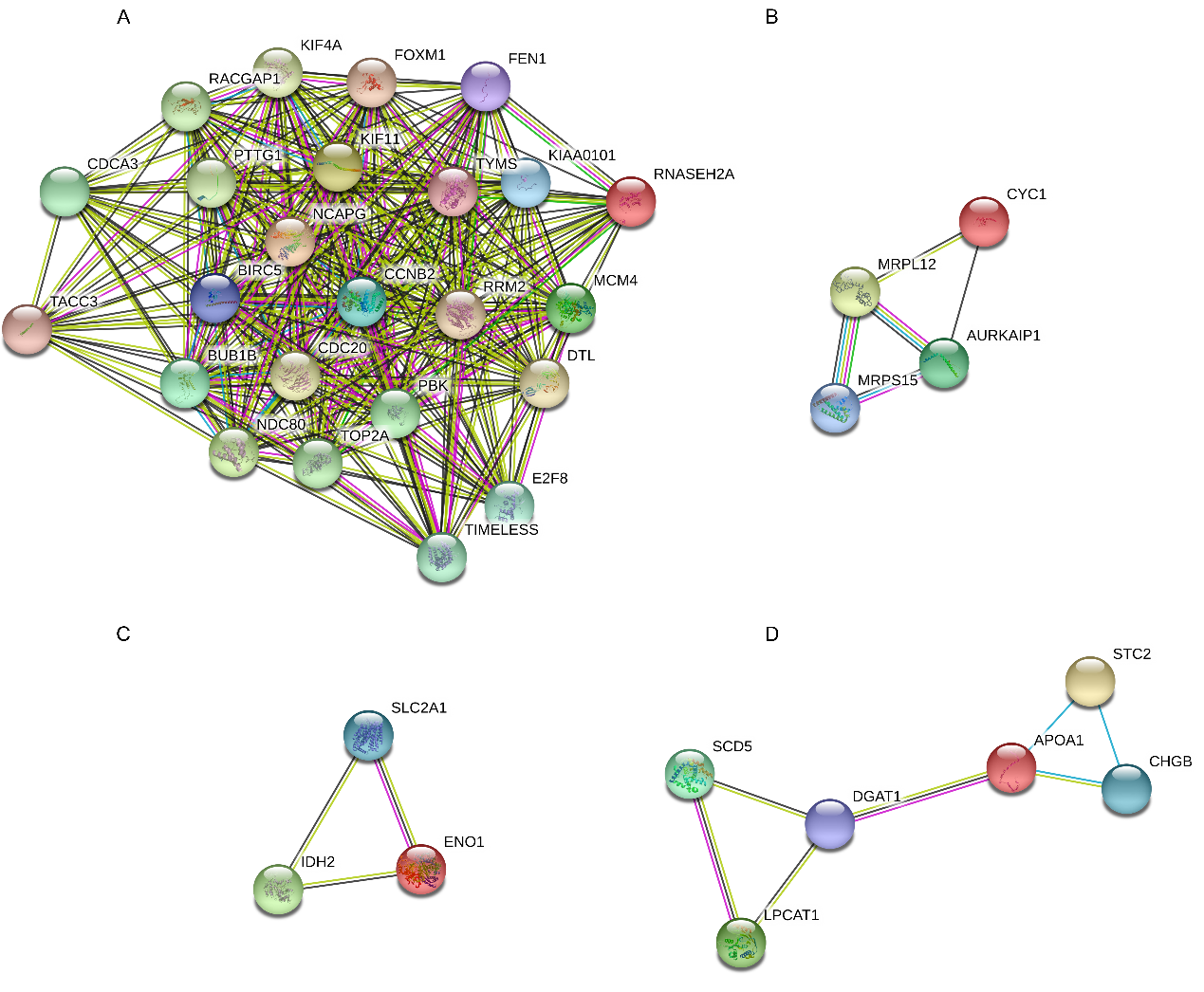
**

(A) Module 1, (B) module 2, (C) module 3, and (D) module 4.
