# Supplementary material for: Integrated bioinformatics analysis for the screening of hub genes and therapeutic drugs in ovarian cancer
Source: J Ovarian Res. 2020 Jan 27;13:10. doi: 10.1186/s13048-020-0613-2 (PMC6986075; doi:10.1186/s13048-020-0613-2)
Supplement: Supplementary file 7 — Additional file 7: KEGG pathway analysis of each module. [file 13048_2020_613_MOESM7_ESM.docx]

**Additional file 7.**

**Table S3. KEGG pathway analysis of each module.**

| Category | Term | Count | P-value | Genes |
| --- | --- | --- | --- | --- |
| Module 1 |  |  |  |  |
| KEGG_PATHWAY | Cell cycle | 5 | <0.001 | CCNB2, BUB1B, CDC20, PTTG1, MCM4 |
| KEGG_PATHWAY | DNA replication | 3 | 0.001 | RNASEH2A, MCM4, FEN1 |
| KEGG_PATHWAY | Oocyte meiosis | 3 | 0.011 | CCNB2, CDC20, PTTG1 |
| Module 2 |  |  |  |  |
| KEGG_PATHWAY | Ribosome | 2 | 0.039 | MRPS15, MRPL12 |
| Module 3 |  |  |  |  |
| KEGG_PATHWAY | Biosynthesis of amino acids | 2 | 0.021 | ENO1, IDH2 |
| KEGG_PATHWAY | HIF-1 signaling pathway | 2 | 0.028 | ENO1, SLC2A1 |
| KEGG_PATHWAY | Carbon metabolism | 2 | 0.033 | ENO1, IDH2 |
| Module 4 |  |  |  |  |
| KEGG_PATHWAY | Fat digestion and absorption | 2 | 0.017 | APOA1, DGAT1 |
| KEGG_PATHWAY | PPAR signaling pathway | 2 | 0.029 | APOA1, SCD5 |
