# Supplementary material for: Integrated bioinformatics analysis for the screening of hub genes and therapeutic drugs in ovarian cancer
Source: J Ovarian Res. 2020 Jan 27;13:10. doi: 10.1186/s13048-020-0613-2 (PMC6986075; doi:10.1186/s13048-020-0613-2)
Supplement: Supplementary file 8 — Additional file 8: Top 10 hub genes in the PPI network ranked by the degree method. [file 13048_2020_613_MOESM8_ESM.docx]

**Additional file 8.**

**Table S4. Top 10 hub genes in the PPI network ranked by the degree method.**

| Rank | Name | Degree Score |
| --- | --- | --- |
| 1 | TYMS | 31 |
| 2 | CCNB2 | 29 |
| 2 | KIF11 | 29 |
| 2 | RRM2 | 29 |
| 5 | CDC20 | 28 |
| 5 | TOP2A | 28 |
| 7 | BUB1B | 27 |
| 8 | BIRC5 | 26 |
| 9 | KIF4A | 25 |
| 9 | FOXM1 | 25 |
