# Supplementary material for: Integrated bioinformatics analysis for the screening of hub genes and therapeutic drugs in ovarian cancer
Source: J Ovarian Res. 2020 Jan 27;13:10. doi: 10.1186/s13048-020-0613-2 (PMC6986075; doi:10.1186/s13048-020-0613-2)
Supplement: Supplementary file 9 — Additional file 9: Progression free survival analyses of the hub genes in early stage (stages 1 and 2) SOC patients. [file 13048_2020_613_MOESM9_ESM.docx]

**Additional file 9.**

**Figure S5.** **Progression free survival analyses of the hub genes in early stage (stages 1 and 2) SOC patients.**

**
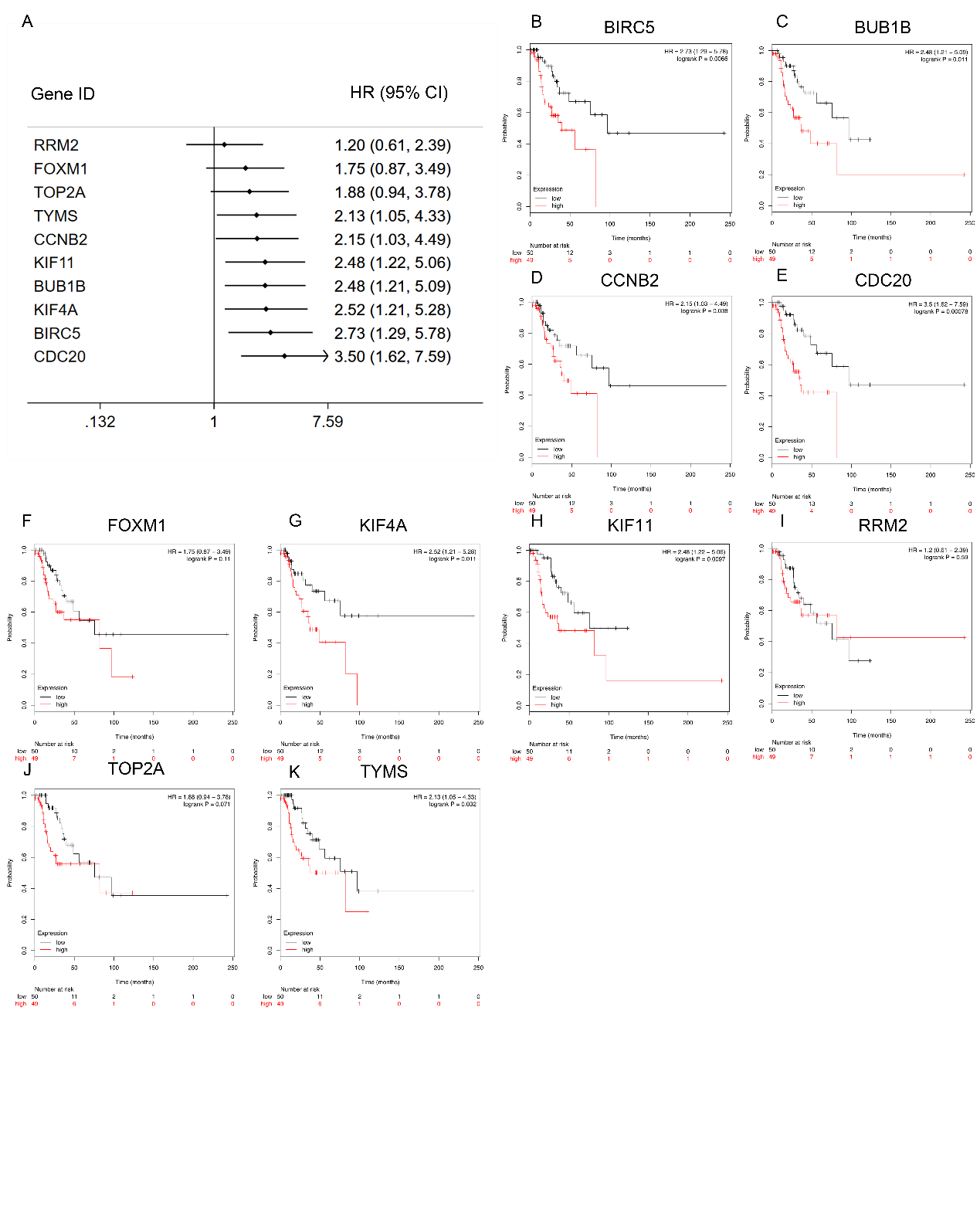
**

(A) Survival prognosis forest map of hub genes related to prognosis in OC patients. Each point in the forest plot represents the hazard ratio (HR) of the gene and the line on both sides of the point represents the 95% confidence interval (95% CI). (B-K) Survival curves constructed by the Kaplan-Meier plotter online database based on the low and high expression of the hub genes in OC patients. Log-rank *P* < 0.05 was considered as statistically significant.
