# Supplementary material for: Integrated bioinformatics analysis for the screening of hub genes and therapeutic drugs in ovarian cancer
Source: J Ovarian Res. 2020 Jan 27;13:10. doi: 10.1186/s13048-020-0613-2 (PMC6986075; doi:10.1186/s13048-020-0613-2)
Supplement: Supplementary file 10 — Additional file 10: Progression free survival analyses of the hub genes in all stage OC patients. [file 13048_2020_613_MOESM10_ESM.docx]

**Additional file 10.**

**Figure S6. Progression free survival analyses of the hub genes in all stage OC patients.**

**
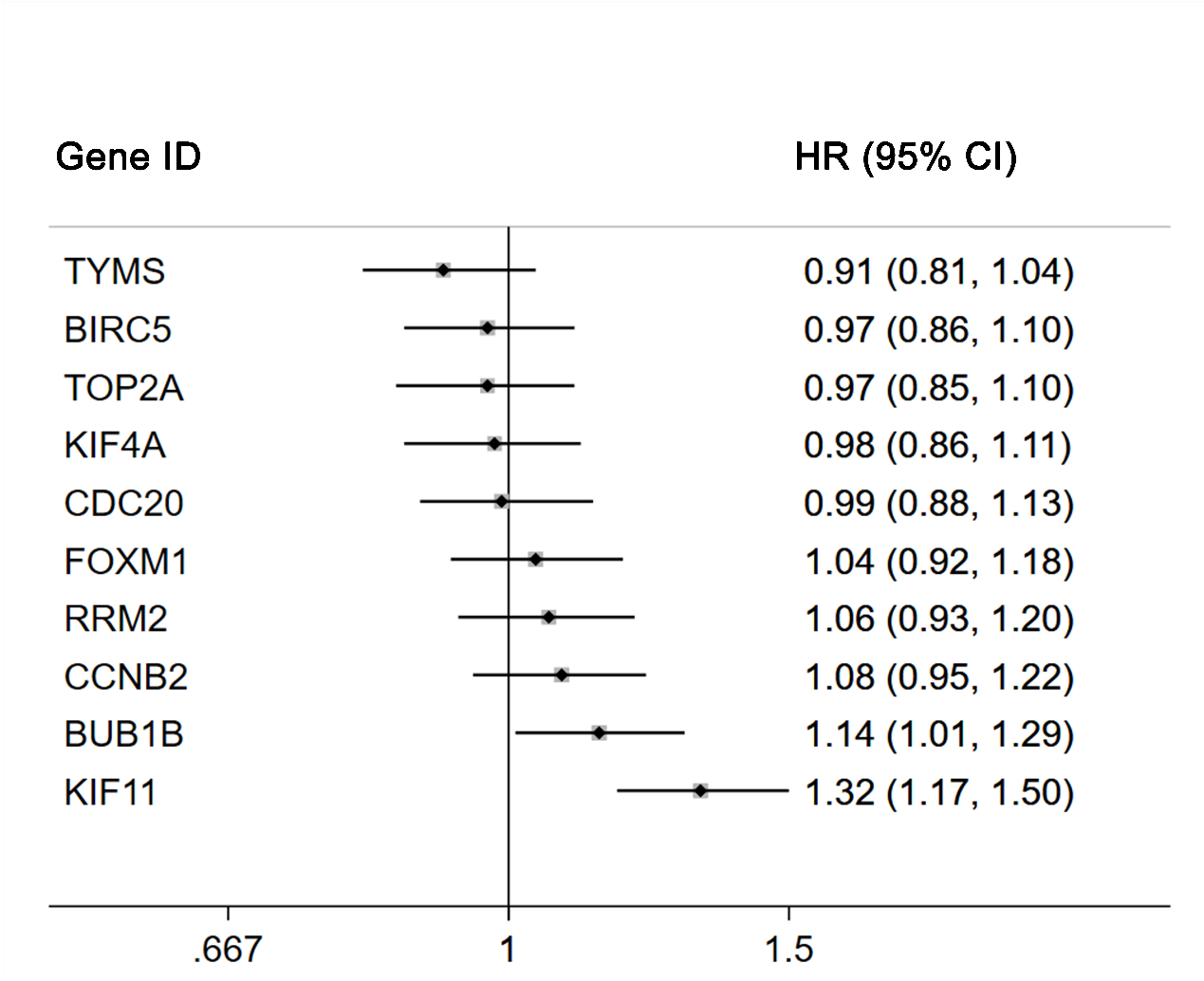
**

Survival prognosis forest map of hub genes related to prognosis in OC patients. Each point in the forest plot represents the hazard ratio (HR) of the gene and the line on both sides of the point represents the 95% confidence interval (95% CI).
