# Supplementary material for: Integrated bioinformatics analysis for the screening of hub genes and therapeutic drugs in ovarian cancer
Source: J Ovarian Res. 2020 Jan 27;13:10. doi: 10.1186/s13048-020-0613-2 (PMC6986075; doi:10.1186/s13048-020-0613-2)
Supplement: Supplementary file 12 — Additional file 12: Progression free survival analyses of the hub genes in advanced stage (stages 3 and 4) OC patients. [file 13048_2020_613_MOESM12_ESM.docx]

**Additional file 12.**

**Figure S8. Progression free survival analyses of the hub genes in advanced stage (stages 3 and 4) OC patients.**

**
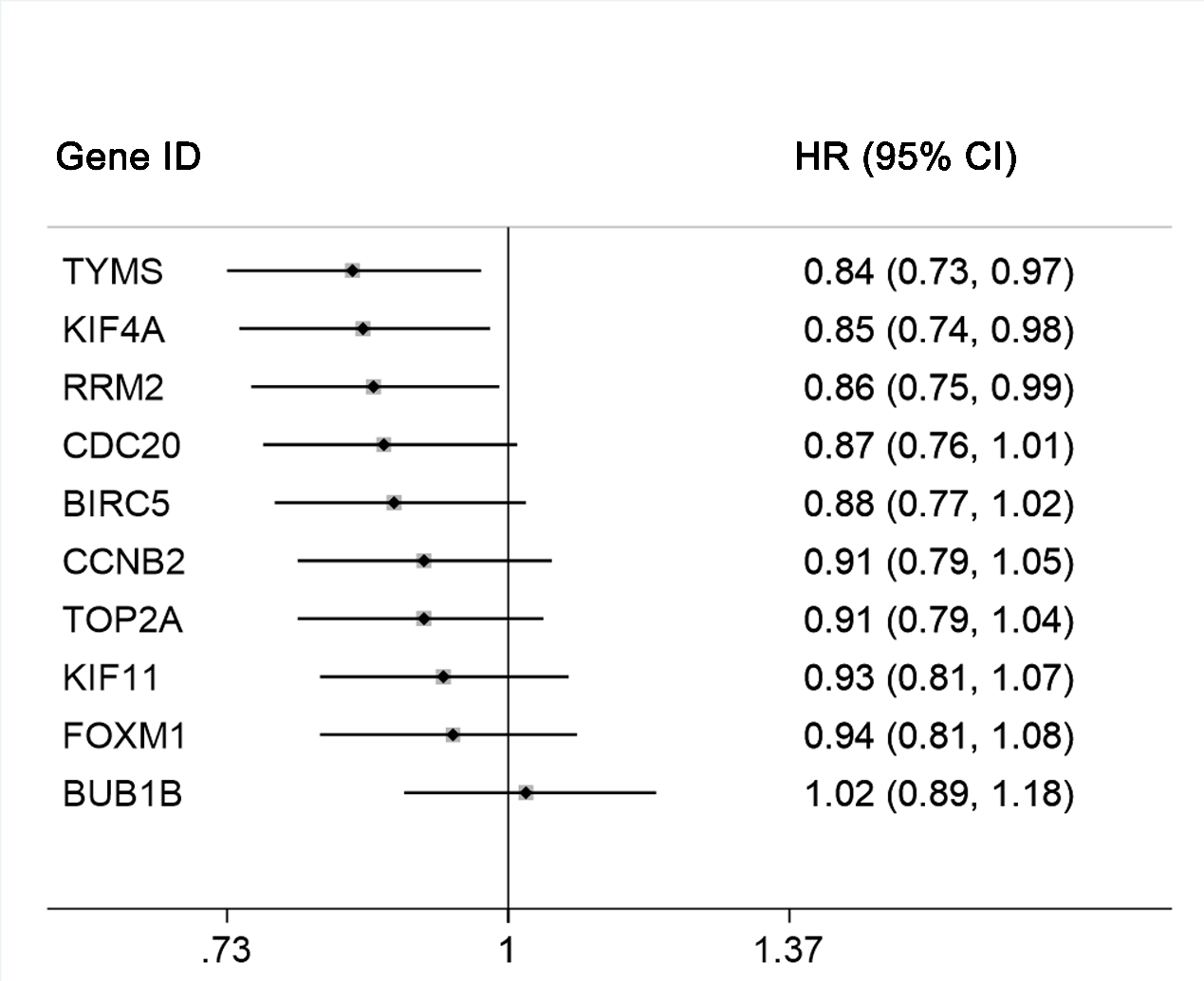
**

Survival prognosis forest map of hub genes related to prognosis in OC patients. Each point in the forest plot represents the hazard ratio (HR) of the gene and the line on both sides of the point represents the 95% confidence interval (95% CI).
