# Supplementary material for: Integrated bioinformatics analysis for the screening of hub genes and therapeutic drugs in ovarian cancer
Source: J Ovarian Res. 2020 Jan 27;13:10. doi: 10.1186/s13048-020-0613-2 (PMC6986075; doi:10.1186/s13048-020-0613-2)
Supplement: Supplementary file 14 — Additional file 14: The respective miRNAs targeting the 10 hub genes. [file 13048_2020_613_MOESM14_ESM.docx]

**Additional file 14.**

**Table S5. The respective miRNAs targeting the 10 hub genes.**

| Genes | miRNAs |
| --- | --- |
| BIRC5 | hsa-miR-182-5p , hsa-miR-218-5p, hsa-miR-128-3p, hsa-miR-135a-5p, hsa-miR-136-5p, hsa-miR-377-3p, hsa-miR-135b-5p, hsa-miR-335-5p, hsa-miR-485-5p, hsa-miR-494-3p, hsa-miR-542-3p, hsa-miR-873-5p |
| BUB1B | hsa-miR-142-3p, hsa-miR-146a-5p, hsa-miR-486-5p, hsa-miR-146b-5p, hsa-miR-875-5p, hsa-miR-543 |
| CCNB2 | hsa-miR-335-5p |
| FOXM1 | hsa-miR-21-5p, hsa-miR-214-3p, hsa-miR-134-5p, hsa-miR-149-5p, hsa-miR-320a, hsa-miR-374a-5p, hsa-miR-342-3p, hsa-miR-494-3p, hsa-miR-590-5p, hsa-miR-876-5p, hsa-miR-877-5p, hsa-miR-374b-5p, hsa-miR-216b-5p, hsa-miR-320b, hsa-miR-320c, hsa-miR-320d |
| KIF4A | hsa-miR-183-5p , hsa-miR-223-3p, hsa-miR-136-5p, hsa-miR-150-5p, hsa-miR-376c-3p, hsa-miR-375, hsa-miR-335-5p, hsa-miR-494-3p, hsa-miR-411-5p, hsa-miR-421, hsa-miR-543 |
| KIF11 | hsa-miR-17-5p , hsa-miR-18a-5p, hsa-miR-20a-5p, hsa-miR-23a-3p, hsa-miR-25-3p, hsa-miR-30a-5p, hsa-miR-32-5p, hsa-miR-92a-3p, hsa-miR-93-5p, hsa-miR-101-3p , hsa-miR-106a-5p, hsa-miR-30c-5p, hsa-miR-30d-5p, hsa-miR-212-3p, hsa-miR-200b-3p, hsa-miR-23b-3p, hsa-miR-30b-5p, hsa-miR-122-5p, hsa-miR-124-3p, hsa-miR-132-3p, hsa-miR-186-5p, hsa-miR-200c-3p, hsa-miR-106b-5p, hsa-miR-30e-5p, hsa-miR-363-3p, hsa-miR-367-3p, hsa-miR-376a-3p, hsa-miR-381-3p, hsa-miR-18b-5p, hsa-miR-20b-5p, hsa-miR-429, hsa-miR-431-5p, hsa-miR-376b-3p, hsa-miR-494-3p, hsa-miR-519d-3p, hsa-miR-506-3p, hsa-miR-92b-3p, hsa-miR-655-3p, hsa-miR-488-3p, hsa-miR-300 |
| RRM2 | hsa-let-7a-5p, hsa-let-7b-5p, hsa-let-7c-5p, hsa-let-7d-5p, hsa-let-7e-5p, hsa-let-7f-5p, hsa-miR-17-5p, hsa-miR-20a-5p, hsa-miR-30a-5p, hsa-miR-31-5p, hsa-miR-93-5p, hsa-miR-106a-5p, hsa-miR-197-3p, hsa-miR-30c-5p, hsa-miR-30d-5p, hsa-miR-204-5p, hsa-miR-211-5p, hsa-let-7g-5p, hsa-let-7i-5p, hsa-miR-30b-5p, hsa-miR-125b-5p, hsa-miR-125a-5p, hsa-miR-106b-5p, hsa-miR-30e-5p, hsa-miR-376c-3p, hsa-miR-377-3p, hsa-miR-342-3p, hsa-miR-20b-5p, hsa-miR-485-5p, hsa-miR-495-3p, hsa-miR-526b-3p, hsa-miR-519d-3p, hsa-miR-874-3p, hsa-miR-4458, hsa-miR-4500 |
| TOP2A | hsa-miR-26a-5p , hsa-miR-26b-5p, hsa-miR-96-5p, hsa-miR-101-3p, hsa-miR-139-5p, hsa-miR-182-5p, hsa-miR-224-5p, hsa-miR-144-3p, hsa-miR-365a-3p, hsa-miR-376c-3p, hsa-miR-377-3p, hsa-miR-383-5p, hsa-miR-335-5p, hsa-miR-329-3p, hsa-miR-410-3p, hsa-miR-485-5p, hsa-miR-495-3p, hsa-miR-411-5p, hsa-miR-28-3p, hsa-miR-543, hsa-miR-1271-5p, hsa-miR-1297 |
| TYMS | hsa-miR-192-5p , hsa-miR-197-3p, hsa-miR-129-5p, hsa-miR-215-5p, hsa-miR-433-3p |
