# Supplementary material for: Integrated bioinformatics analysis for the screening of hub genes and therapeutic drugs in ovarian cancer
Source: J Ovarian Res. 2020 Jan 27;13:10. doi: 10.1186/s13048-020-0613-2 (PMC6986075; doi:10.1186/s13048-020-0613-2)
Supplement: Supplementary file 15 — Additional file 15: Targetable TYMS and BIRC5 subnetwork. [file 13048_2020_613_MOESM15_ESM.docx]

**Additional file 15.**

**Figure S10. Targetable TYMS and BIRC5 subnetwork.**

**
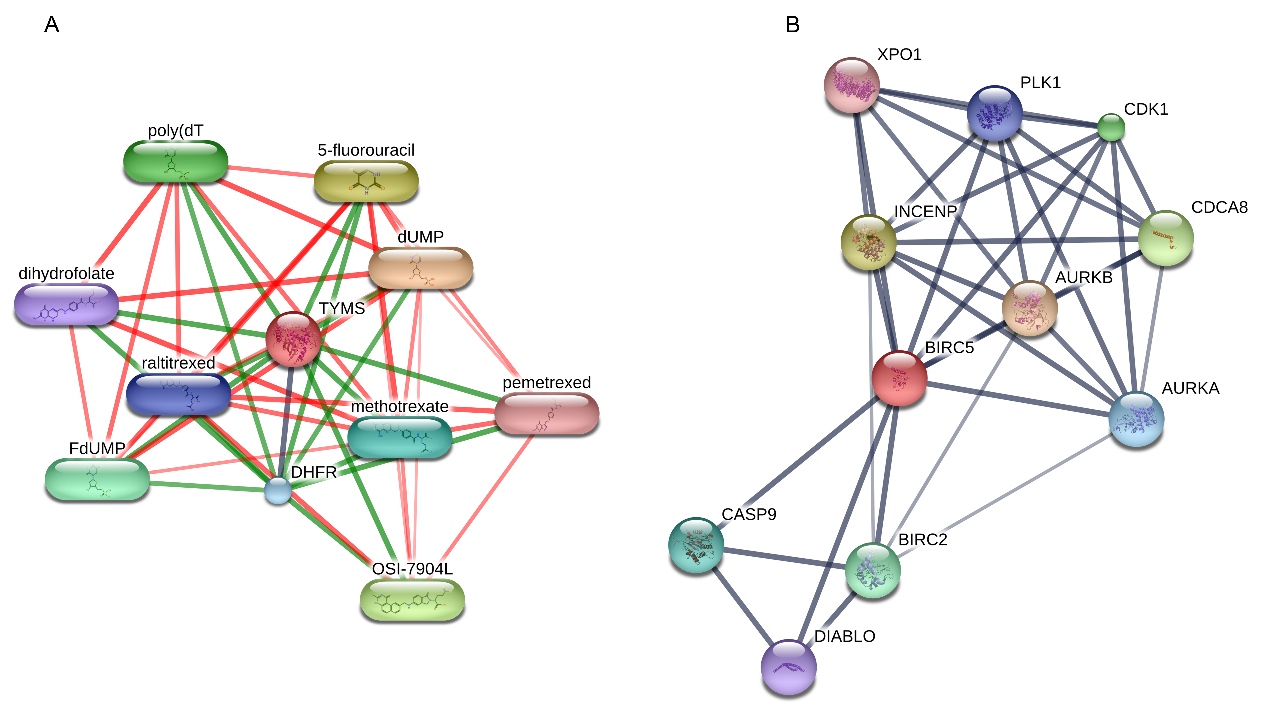
**

(A) Subnetwork of TYMS performed by the STITCH online database contains 2 nodes, 1 edge, and its *P-value* of PPI enrichment analysis was 0.506. (B) Subnetwork of BIRC5 performed by the STITCH online database contains 11 nodes and 36 edges, and its *P-value* of PPI enrichment analysis was 1.70e-06.
